# Supplementary material for: Amido-Bridged Nucleic Acid-Modified Antisense Oligonucleotides Targeting SYT13 to Treat Peritoneal Metastasis of Gastric Cancer
Source: Mol Ther Nucleic Acids. 2020 Oct 6;22:791–802. doi: 10.1016/j.omtn.2020.10.001 (PMC7644579; doi:10.1016/j.omtn.2020.10.001)
Supplement: Document S1. Figures S1–S6, Tables S1–S3, and Supplemental Materials and Methods [file mmc1.pdf]

OMTN, Volume *22*

Supplemental Information

Amido-Bridged Nucleic Acid-Modified Antisense Oligonucleotides Targeting *SYT13* to Treat Peritoneal Metastasis of Gastric Cancer

Mitsuro Kanda, Yuuya Kasahara, Dai Shimizu, Takashi Miwa, Shinichi Umeda, Koichi Sawaki, Shunsuke Nakamura, Yasuhiro Kodera, and Satoshi Obika

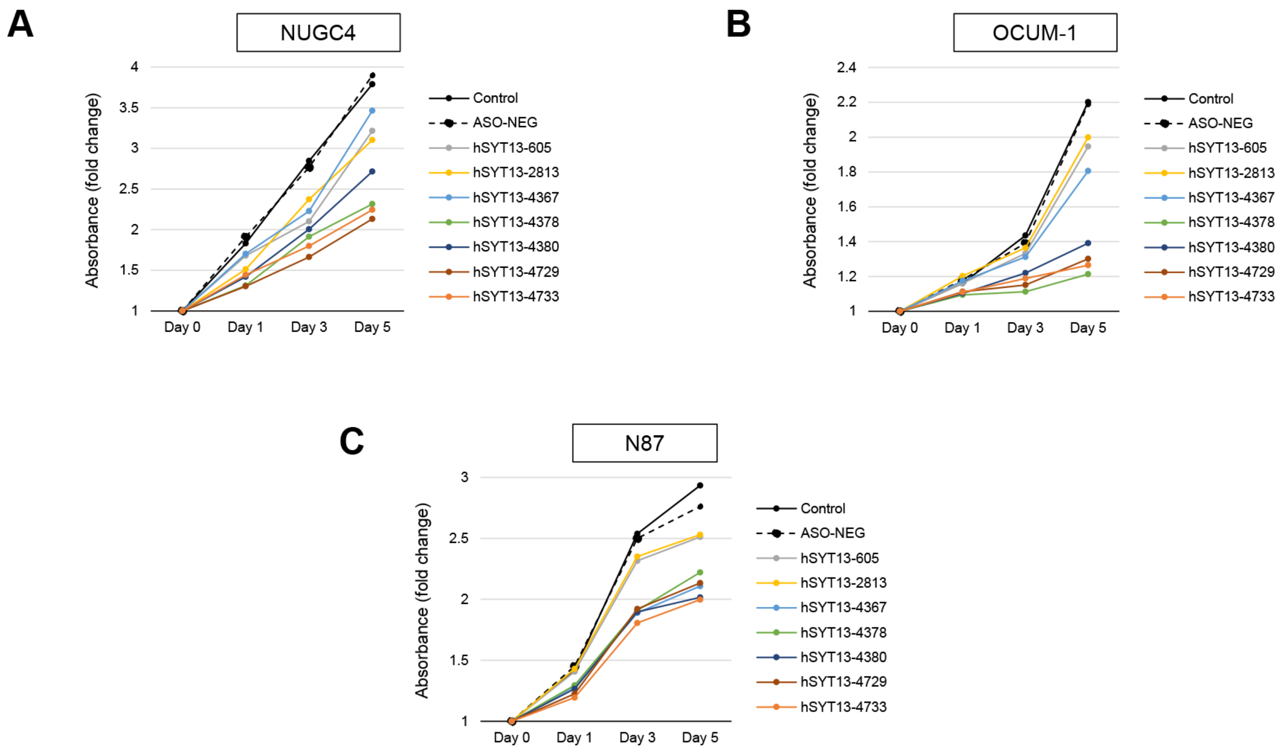

Figure S1. Comparison of effects of ASOs on the proliferation of the gastric cancer cell lines NUGC4, OCUM-1, and N87.

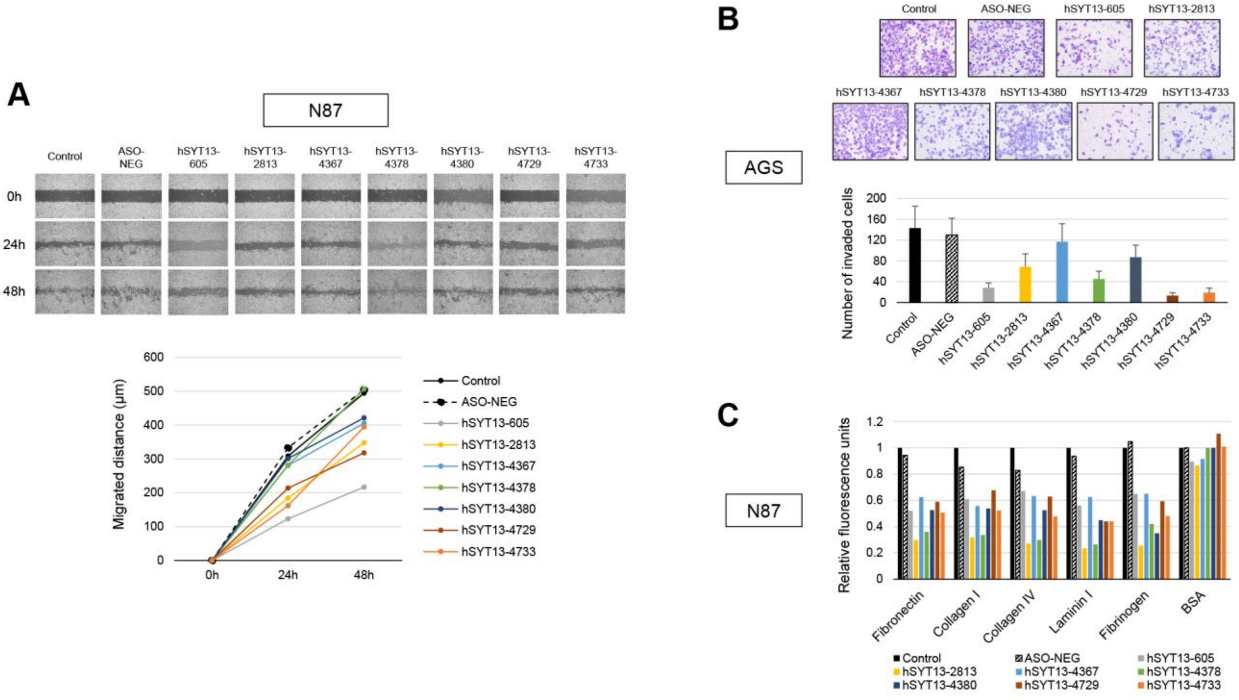

Figure S2. Comparison of effects of ASOs on the migration (*A*), invasiveness (*B*), and adhesion (*C*) of gastric cancer cells.

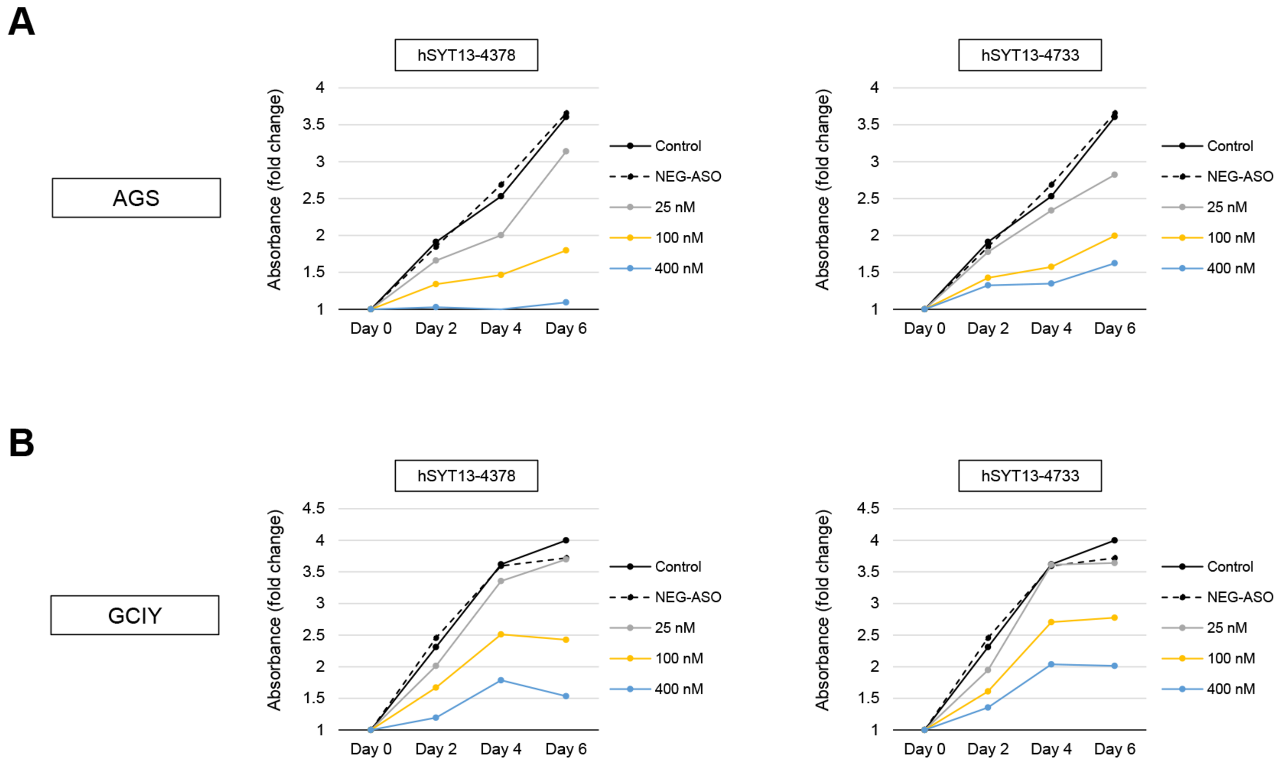

Figure S3. Concentration-dependent in vitro inhibitory effects of AmNA-modified anti-SYT13 ASOs on proliferation of AGS (A) and GCIY (B) cells.

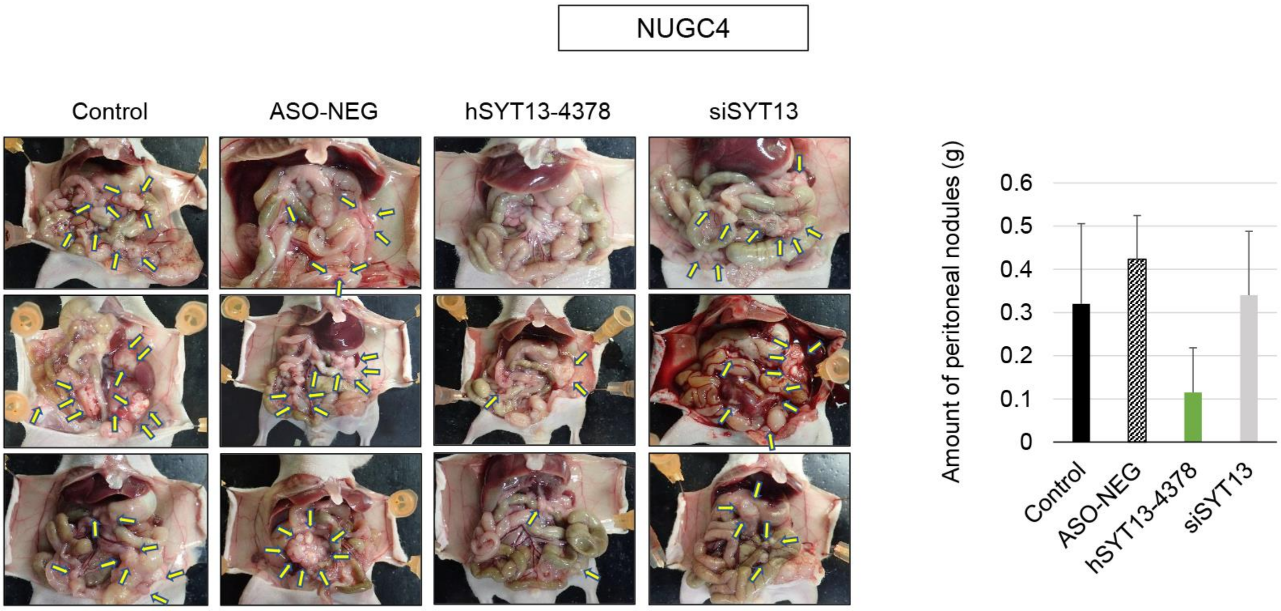

Figure S4. Therapeutic effects of intraperitoneal administration of hSYT13-4378. Macroscopic appearance of peritoneal nodules 6 weeks after implantation of NUGC4 cells and comparison of the total volume of peritoneal nodules of each treatment group.

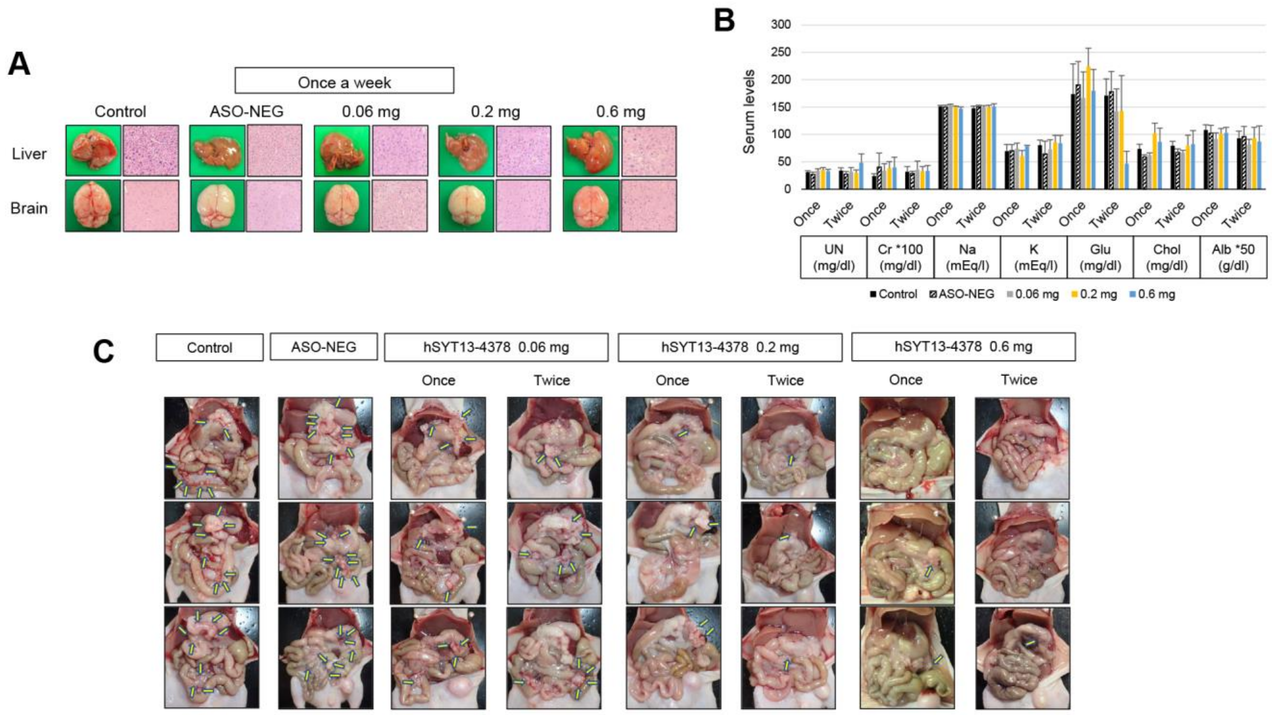

Figure S5. Toxicity to mice of intraperitoneally administered AmNA-modified anti-SYT13 ASOs. (*A*) Renal functions and metabolic parameters. (*B*) Macroscopic and microscopic findings of the brain and liver the once weekly-treated groups. (*C*) Macroscopic appearance of peritoneal nodules of each treatment group.

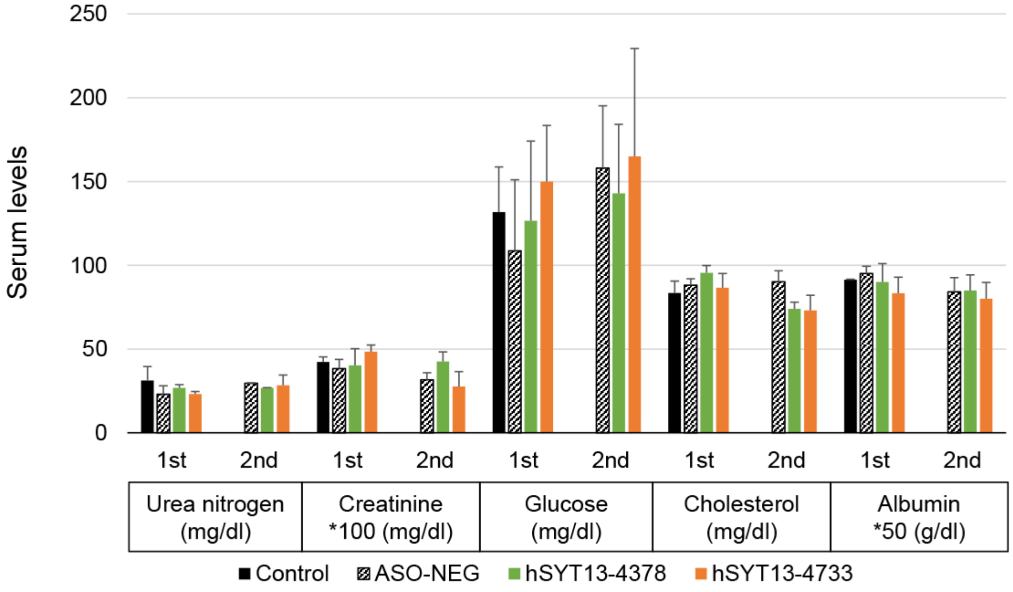

Figure S6. Blood tests after administration of 0.2 mg hSYT13-4378 or hSYT13-4733 twice weekly for 2 weeks and 2 weeks after treatment ceased.

Table S1. Sequences of 71 candidate amido-bridged nucleic acid-modified antisense oligonucleotides targeting human *SYT13* and a negative-control antisense oligonucleotide

Name	Length	Sequence
hSYT13-350-AmNA	15-mer	G(Y)^G(Y)^A(Y)^a^c^t^t^g^a^g^g^a^G(Y)^G(Y)^g
hSYT13-601-AmNA	17-mer	T(Y)^A(Y)^G(Y)^t^g^g^a^g^t^t^t^g^g^g^G(Y)^G(Y)^c
hSYT13-603-AmNA	15-mer	T(Y)^A(Y)^G(Y)^t^g^g^a^g^t^t^t^g^G(Y)^G(Y)^g
hSYT13-603-AmNA	17-mer	A(Y)^G(Y)^T(Y)^a^g^t^g^g^a^g^t^t^t^g^G(Y)^G(Y)^g
hSYT13-605-AmNA	15-mer	A(Y)^G(Y)^T(Y)^a^g^t^g^g^a^g^t^t^T(Y)^G(Y)^g
hSYT13-605-AmNA	17-mer	G(Y)^5(Y)^A(Y)^g^t^a^g^t^g^g^a^g^t^t^T(Y)^G(Y)^g
hSYT13-607-AmNA	13-mer	A(Y)^G(Y)^T(Y)^a^g^t^g^g^a^g^T(Y)^T(Y)^t
hSYT13-607-AmNA	15-mer	G(Y)^5(Y)^A(Y)^g^t^a^g^t^g^g^a^g^T(Y)^T(Y)^t
hSYT13-607-AmNA	17-mer	A(Y)^G(Y)^G(Y)^c^a^g^t^a^g^t^g^g^a^g^T(Y)^T(Y)^t
hSYT13-609-AmNA	13-mer	G(Y)^5(Y)^A(Y)^g^t^a^g^t^g^g^A(Y)^G(Y)^t
hSYT13-609-AmNA	15-mer	A(Y)^G(Y)^G(Y)^c^a^g^t^a^g^t^g^g^A(Y)^G(Y)^t
hSYT13-609-AmNA	17-mer	5(Y)^5(Y)^A(Y)^g^g^c^a^g^t^a^g^t^g^g^A(Y)^G(Y)^t
hSYT13-999-AmNA	15-mer	5(Y)^T(Y)^G(Y)^a^t^g^g^a^t^a^g^t^A(Y)^G(Y)^g
hSYT13-1000-AmNA	15-mer	G(Y)^5(Y)^T(Y)^g^a^t^g^g^a^t^a^g^T(Y)^A(Y)^g
hSYT13-1071-AmNA	15-mer	T(Y)^T(Y)^G(Y)^g^a^c^t^g^g^t^t^a^G(Y)^A(Y)^g
hSYT13-1072-AmNA	15-mer	5(Y)^T(Y)^T(Y)^g^g^a^c^t^g^g^t^t^A(Y)^G(Y)^a
hSYT13-1421-AmNA	15-mer	5(Y)^5(Y)^A(Y)^a^g^a^a^g^g^g^a^g^G(Y)^5(Y)^a
hSYT13-1614-AmNA	15-mer	A(Y)^5(Y)^A(Y)^g^a^t^g^a^g^c^a^a^A(Y)^A(Y)^t
hSYT13-1617-AmNA	15-mer	A(Y)^A(Y)^5(Y)^a^c^a^g^a^t^g^a^g^5(Y)^A(Y)^a
hSYT13-1618-AmNA	15-mer	T(Y)^A(Y)^A(Y)^c^a^c^a^g^a^t^g^a^G(Y)^5(Y)^a
hSYT13-1619-AmNA	15-mer	A(Y)^T(Y)^A(Y)^a^c^a^c^a^g^a^t^g^A(Y)^G(Y)^c
hSYT13-1622-AmNA	15-mer	T(Y)^5(Y)^A(Y)^a^t^a^a^c^a^c^a^g^A(Y)^T(Y)^g
hSYT13-1623-AmNA	15-mer	T(Y)^T(Y)^5(Y)^a^a^t^a^a^c^a^c^a^G(Y)^A(Y)^t
hSYT13-1625-AmNA	15-mer	5(Y)^5(Y)^T(Y)^t^c^a^a^t^a^a^c^a^5(Y)^A(Y)^g
hSYT13-1777-AmNA	15-mer	G(Y)^A(Y)^T(Y)^a^c^t^t^t^c^a^c^t^T(Y)^5(Y)^c
hSYT13-2631-AmNA	15-mer	A(Y)^A(Y)^G(Y)^g^c^t^c^a^t^a^a^t^T(Y)^T(Y)^a
hSYT13-2812-AmNA	15-mer	T(Y)^T(Y)^T(Y)^t^t^a^g^c^c^a^g^a^G(Y)^A(Y)^g
hSYT13-2813-AmNA	15-mer	G(Y)^T(Y)^T(Y)^t^t^t^a^g^c^c^a^g^A(Y)^G(Y)^a
hSYT13-2814-AmNA	15-mer	T(Y)^G(Y)^T(Y)^t^t^t^t^a^g^c^c^a^G(Y)^A(Y)^g
hSYT13-2815-AmNA	15-mer	T(Y)^T(Y)^G(Y)^t^t^t^t^t^a^g^c^c^A(Y)^G(Y)^a
hSYT13-3246-AmNA	15-mer	5(Y)^5(Y)^A(Y)^a^a^g^g^c^a^g^a^a^T(Y)^5(Y)^c
hSYT13-3317-AmNA	15-mer	A(Y)^A(Y)^T(Y)^t^c^t^g^t^c^t^t^a^G(Y)^G(Y)^g
hSYT13-3425-AmNA	15-mer	G(Y)^A(Y)^A(Y)^a^a^a^a^t^a^a^t^g^A(Y)^5(Y)^c
hSYT13-3426-AmNA	15-mer	A(Y)^G(Y)^A(Y)^a^a^a^a^a^t^a^a^t^G(Y)^A(Y)^c
hSYT13-4268-AmNA	15-mer	T(Y)^T(Y)^T(Y)^a^c^c^a^t^t^g^a^g^A(Y)^A(Y)^c

hSYT13-4330-AmNA	15-mer	T(Y)^A(Y)^G(Y)^g^c^a^a^a^t^a^g^t^A(Y)^A(Y)^t
hSYT13-4367-AmNA	15-mer	G(Y)^T(Y)^T(Y)^g^a^t^t^a^c^a^t^t^T(Y)^A(Y)^c
hSYT13-4368-AmNA	15-mer	T(Y)^G(Y)^T(Y)^t^g^a^t^t^a^c^a^t^T(Y)^T(Y)^a
hSYT13-4371-AmNA	15-mer	A(Y)^T(Y)^5(Y)^t^g^t^t^g^a^t^t^a^5(Y)^A(Y)^t
hSYT13-4373-AmNA	15-mer	T(Y)^5(Y)^A(Y)^t^c^t^g^t^t^g^a^t^T(Y)^A(Y)^c
hSYT13-4374-AmNA	13-mer	5(Y)^A(Y)^T(Y)^c^t^g^t^t^g^a^T(Y)^T(Y)^a
hSYT13-4374-AmNA	15-mer	T(Y)^T(Y)^5(Y)^a^t^c^t^g^t^t^g^a^T(Y)^T(Y)^a
hSYT13-4374-AmNA	17-mer	T(Y)^5(Y)^T(Y)^t^c^a^t^c^t^g^t^t^g^a^T(Y)^T(Y)^a
hSYT13-4374-AmNA	19-mer	T(Y)^5(Y)^T(Y)^c^t^t^c^a^t^c^t^g^t^t^g^a^T(Y)^T(Y)^a
hSYT13-4376-AmNA	15-mer	T(Y)^5(Y)^T(Y)^t^c^a^t^c^t^g^t^t^G(Y)^A(Y)^t
hSYT13-4376-AmNA	17-mer	T(Y)^5(Y)^T(Y)^c^t^t^c^a^t^c^t^g^t^t^G(Y)^A(Y)^t
hSYT13-4377-AmNA	15-mer	5(Y)^T(Y)^5(Y)^t^t^c^a^t^c^t^g^t^T(Y)^G(Y)^a
hSYT13-4378-AmNA	15-mer	T(Y)^5(Y)^T(Y)^c^t^t^c^a^t^c^t^g^T(Y)^T(Y)^g
hSYT13-4378-AmNA	17-mer	A(Y)^T(Y)^T(Y)^c^t^c^t^t^c^a^t^c^t^g^T(Y)^T(Y)^g
hSYT13-4380-AmNA	15-mer	A(Y)^T(Y)^T(Y)^c^t^c^t^t^c^a^t^c^T(Y)^G(Y)^t
hSYT13-4380-AmNA	17-mer	A(Y)^T(Y)^A(Y)^t^t^c^t^c^t^t^c^a^t^c^T(Y)^G(Y)^t
hSYT13-4381-AmNA	15-mer	T(Y)^A(Y)^T(Y)^t^c^t^c^t^t^c^a^t^5(Y)^T(Y)^g
hSYT13-4382-AmNA	15-mer	A(Y)^T(Y)^A(Y)^t^t^c^t^c^t^t^c^a^T(Y)^5(Y)^t
hSYT13-4382-AmN	17-mer	T(Y)^T(Y)^A(Y)^t^a^t^t^c^t^c^t^t^c^a^T(Y)^5(Y)^t
hSYT13-4716-AmNA	15-mer	5(Y)^5(Y)^5(Y)^g^a^t^t^t^t^c^t^a^T(Y)^5(Y)^c
hSYT13-4717-AmNA	15-mer	T(Y)^5(Y)^5(Y)^c^g^a^t^t^t^t^c^t^A(Y)^T(Y)^c
hSYT13-4725-AmNA(	13-mer	G(Y)^5(Y)^A(Y)^g^a^c^t^c^c^c^G(Y)^A(Y)^t
hSYT13-4725-AmN	15-mer	G(Y)^G(Y)^G(Y)^c^a^g^a^c^t^c^c^c^G(Y)^A(Y)^t
hSYT13-4725-AmN	17-mer	G(Y)^A(Y)^G(Y)^g^g^c^a^g^a^c^t^c^c^c^G(Y)^A(Y)^t
hSYT13-4727-AmNA	13-mer	G(Y)^G(Y)^G(Y)^c^a^g^a^c^t^c^5(Y)^5(Y)^g
hSYT13-4727-AmNA	15-mer	G(Y)^A(Y)^G(Y)^g^g^c^a^g^a^c^t^c^5(Y)^5(Y)^g
hSYT13-4727-AmNA	17-mer	A(Y)^T(Y)^G(Y)^a^g^g^g^c^a^g^a^c^t^c^5(Y)^5(Y)^g
hSYT13-4729-AmNA	15-mer	A(Y)^T(Y)^G(Y)^a^g^g^g^c^a^g^a^c^T(Y)^5(Y)^c
hSYT13-4778-AmNA	15-mer	G(Y)^5(Y)^T(Y)^c^a^a^c^a^a^a^t^a^G(Y)^A(Y)^t
hSYT13-4779-AmNA	15-mer	T(Y)^G(Y)^5(Y)^t^c^a^a^c^a^a^a^t^A(Y)^G(Y)^a
hSYT13-4729-AmNA	17-mer	T(Y)^A(Y)^A(Y)^t^g^a^g^g^g^c^a^g^a^c^T(Y)^5(Y)^c
hSYT13-4731-AmNA	15-mer	T(Y)^A(Y)^A(Y)^t^g^a^g^g^g^c^a^g^A(Y)^5(Y)^t
hSYT13-4731-AmNA	17-mer	A(Y)^T(Y)^T(Y)^a^a^t^g^a^g^g^g^c^a^g^A(Y)^5(Y)^t
hSYT13-4733-AmNA	17-mer	A(Y)^G(Y)^A(Y)^t^t^a^a^t^g^a^g^g^g^c^A(Y)^G(Y)^a
hSYT13-4951-AmNA	15-mer	5(Y)^A(Y)^5(Y)^a^t^t^t^t^a^c^c^c^A(Y)^G(Y)^g
hSYT13-4952-AmNA	15-mer	5(Y)^5(Y)^A(Y)^c^a^t^t^t^t^a^c^c^5(Y)^A(Y)^g
NEG-ASO	15-mer	5(Y)^A(Y)^5(Y)^a^g^t^a^t^c^t^a^t^G(Y)^T(Y)^a

Table S2. Genes at potential risk of off-target effects of amido-bridged nucleic acid-modified antisense oligonucleotides targeting human *SYT13*

**Antisense oligonucleotides**	**Off-target result**	**mRNA accession number**	**Transcription product**
hSYT13-4378	Full match	Not identified	
	1 mismatch	NM_003772.3	JRK like, transcript variant 1
		NR_104262.1	cell division cycle and apoptosis regulator 1, transcript variant 4
	2 mismatches	NM_000189.4	hexokinase 2
		NM_001009612.2	chromosome 20 open reading frame 202
		NM_001035.2	ryanodine receptor 2
		NM_001145365.1	zinc finger protein 652, transcript variant 1
		NM_001247997.1	CAP-Gly domain containing linker protein 1, transcript variant 3
		NM_001252613.1	zinc finger protein 638, transcript variant 4
		NM_001286206.1	centrosomal protein 162, transcript variant 2
		NM_001999.3	fibrillin 2
		NM_005546.3	IL2 inducible T cell kinase
		NM_006726.4	LPS responsive beige-like anchor protein, transcript variant 2
		NM_018313.4	polybromo 1, transcript variant 2
		NM_024636.3	STEAP4 metalloreductase, transcript variant 1
		NM_130759.3	GTPase, IMAP family member 1
		NM_173493.2	PAS domain containing repressor 1
		NR_027902.1	long intergenic non-protein coding RNA 303
		NR_033857.1	BMS1 pseudogene 21
		NR_037804.1	NPHP3-ACAD11 readthrough
		XM_011534442.1	CREB3 regulatory factor, transcript variant X3
		XR_245956.1	nuclear receptor subfamily 2 group C member 1, transcript variant X10
hSYT13-4733	Full match	Not identified	
	1 mismatch	Not identified	
	2 mismatches	XR_429721.2	pyruvate dehydrogenase phosphatase regulatory subunit, transcript variant X10

Table S3. Antibodies used for digital western blot imaging analyses

Target	Predicted size (kDa)	Antibody type	Company	Catalog number	Dilution
SYT13	47	Rabbit polyclonal	Aviva Systems Biology Corporation	OAAB02896	1:50
FAK	125	Rabbit monoclonal	Cell Signaling Technology	#71433	1:50
p-FAK	125	Rabbit monoclonal	Cell Signaling Technology	#8556	1:50
AKT	60	Rabbit monoclonal	Cell Signaling Technology	#4691	1:50
p-AKT	60	Rabbit monoclonal	Cell Signaling Technology	#4060	1:50
GSK3B	46	Rabbit monoclonal	Cell Signaling Technology	#12456	1:50
p-GSK3B	46	Rabbit monoclonal	Cell Signaling Technology	#5558	1:50
JAK2	125	Rabbit monoclonal	Cell Signaling Technology	#3230	1:50
p-JAK2	125	Rabbit monoclonal	Cell Signaling Technology	#3776	1:50
YAP	65-75	Rabbit monoclonal	Cell Signaling Technology	#14074	1:50
p-YAP(ser109)	78	Rabbit monoclonal	Cell Signaling Technology	#46931	1:50
Bad	23	Rabbit monoclonal	Cell Signaling Technology	#9239	1:50
p-Bad	23	Rabbit monoclonal	Cell Signaling Technology	#5284	1:50
BIRC5	16	Rabbit monoclonal	Cell Signaling Technology	#2808	1:50
CDK2	33	Rabbit monoclonal	Cell Signaling Technology	#2546	1:50
p-CDK2	33	Rabbit monoclonal	Cell Signaling Technology	#2561	1:50
b-actin	42	Mouse monoclonal	Abcam	ab8224	1:50

Supplemental Materials and Methods

Clinical Significance of *SYT13* Expression

Immunohistochemical (IHC) analysis of the in situ location and expression patterns of SYT13 was performed using a rabbit polyclonal antibody raised against SYT13 (OAAB02896; Aviva Systems Biology Corporation, San Diego, CA, USA) diluted 1: 100 in antibody diluent (Dako, Glostrup, Denmark) to probe 40 formalin-fixed, paraffin-embedded sections of well-preserved tissues from patients with pT4a gastric cancer who underwent curative gastrectomy, as previously described.[1] The staining intensity of SYT13 was categorized as negative or positive according to the percentage of stained cells in a gastric cancer component (negative, 0%–20%; positive, >20%).[2, 3] To avoid subjectivity, specimens were randomized and coded by two independent observers masked to the status of the samples.

External-validation cohorts were generated using global cohort data obtained from The Cancer Genome Atlas (TCGA) Research Network via the open source c-BioPortal (https://www.cbioportal.org/)[4] and the Kaplan–Meier Plotter (http://kmplot.com/analysis/).[4]

Cell Lines

The gastric cancer cell lines MKN1 (JCRB1433), MKN74 (JCRB0255), NUGC4 (JCRB0834), and GCIY (JRCB0555) were obtained from the Japanese Collection of Research Bioresources Cell Bank

(JCRB, Osaka, Japan). The AGS (CRL1739), KATO-III (HTB103) and N87 (CRL5822) cell lines were acquired from the American Type Culture Collection (ATCC, Manassas, VA, USA). The cell lines were tested using the short tandem-repeat PCR method and authenticated by the JCRB before the study commenced (June 2015). Cell lines were cultured at 37 °C in RPMI 1640 medium (Sigma–Aldrich) supplemented with 10% fetal bovine serum (FBS) in an atmosphere containing 5% CO_2.

Design and Synthesis of AmNA-modified Anti-SYT13 ASOs

The loop structure of *SYT13* mRNA, to which ASOs binds with high affinity, was predicted using RNAfold (http://rna.tbi.univie.ac.at//cgi-bin/RNAWebSuite/RNAfold.cgi) and mfold (http://unafold.rna.albany.edu/?q=mfold).[5] Effective sequences specifically targeting human *SYT13* mRNA were selected in consideration of homology with mus musculus for the analysis of in vivo toxicity. We designed AmNA-modified ASOs to exclude the sequences TCC and TGC, which may bind hepatocellular proteins to cause hepatotoxicity.[6, 7] CpG, which strongly induces strong inflammatory responses, was avoided as well.[8] AmNA-modified ASOs were synthesized and purified by Gene Design, Inc. using an automated DNA synthesizer (Osaka, Japan).

In Vitro Transfection of ASOs and siRNA

Transfection of ASOs or siRNA into gastric cancer cell lines was performed using the CEM method. Cells were cultured in a 24-well plate (5000 cells per well) and transiently transfected the next day

with AmNA-modified ASOs (6.25–400 nmol/L) or siRNAs specific for *SYT13* (100 nmol/L (A-014082-13 and A-014082-14) (Accell siRNA; GE Healthcare Dharmacon, Lafayette, CO, USA) in the presence of 9 mM $CaCl_2$. After transfection, cells were cultured in medium with 10% FBS for 24 h. The efficacy of inhibition of *SYT13* mRNA expression was evaluated using a quantitative real-time reverse-transcription PCR (qRT-PCR) assay with a StepOnePlus Real-Time PCR System (Applied Biosystems, Foster City, CA, USA). The *SYT13* primers were as follows: sense 5′-TGGTGGTGCTGATTAAAGCC -3′ and antisense 5′- TGCTTCTTCTTCAGCTTCCG -3′. Glyceraldehyde-3-phosphate dehydrogenase (*GAPDH*) mRNA served as an endogenous control.

Assays of Cell Function

A Cell Counting Kit-8 (Dojindo Molecular Technologies, Inc., Kumamoto, Japan) was used to analyze cell proliferation. Wound-healing assays were performed to evaluate cell migration, and cell invasiveness was assessed using BioCoat Matrigel invasion chambers (BD Biosciences, Bedford, MA, USA). We used the CytoSelect 48-Well Cell Adhesion Assay (Cell Biolabs, Inc., San Diego, CA, USA) to measure the adherence of cells to extracellular matrix components. These assays were performed as previously described.[9, 10] To evaluate total caspase activity, a Muse MultiCaspase Kit (Merck Millipore, Billerica, MA, USA) was used, and data analysis was performed using a Muse Cell Analyzer (Merck Millipore). The activities of caspase-3, 8, 9, and 12 were measured using the Caspase Colorimetric Assay Kit (BioVision, Milpitas, CA, USA). Mitochondrial membrane potential

and cell-cycle distribution were assessed using a Muse MitoPotential Kit (Merck Millipore) and a Muse Cell Cycle Kit (Merck Millipore), respectively.

Assays of Cancer Cell Stemness

Aldehyde dehydrogenase (ALDH), a surrogate marker of stem/progenitor cells, was estimated using the ALDEFLOUR fluorescent reagent system (Stem Cell Technologies, Vancouver, BC, Canada). Briefly, gastric cancer cells (10^5/ml) were suspended in ALDEFLOUR buffer. The ALDH inhibitor N,N-diethylaminobenzaldehyde (25 μM) was added to cells that were then incubated with the activated ALDEFLOUR substrate boron-dipyrromethene aminoacetaldehyde (1.5 μM) for 45 min at 37 °C. The proportion of ALDH-positive cells was determined using a FACS Calibur system (BD Biosciences, Franklin Lakes, NJ, USA), and data were analyzed using CellQuestPro software (BD Biosciences). The three-dimensional-spheroid cultures were analyzed using PrimeSurface96U multiwell plates (Sumitomo Bakelite, Tokyo, Japan). MKN1 cells were mock-transfected or transfected with a nontargeting control ASO (ASO-NEG) or AmNA-modified anti-SYT13 ASOs (hSYT13-4378 and hSYT13-4733) and incubated for 48 h. Each well contained 5000 cells. After incubation for 72 h, images of spheroids were acquired using an FSX100 (Olympus, Tokyo, Japan) fluorescence microscope.

***SYT13* Expression Vector**

SYT13 cDNAs were ligated to the pFN21A HaloTag CMV Flexi Vector (Promega, Madison, WI, USA). The *SYT13* vector (0.2 mg) was used to transfect MKN74 cells (1×10^5) using the NEON Transfection System (Thermo Fisher Scientific, Waltham, MA, USA).

Microarray and In Silico Analysis of Candidate Genes for Potential Off-target Effects of AmNA-modified Anti-SYT13 ASOs

Total RNAs extracted from NUGC4 cells transfected with mock, ASO-NEG, or AmNA-modified anti-SYT13 ASOs (hSYT13-4378 and hSYT13-4733) were subjected to microarray analysis using a 3D-Gene microarray (Toray, Tokyo, Japan). Global gene expression data were selected to evaluate potential off-target effects of AmNA-modified Anti-SYT13 ASOs as follows: 1) ≥2-fold increase or ≤0.5-fold decrease compared with mock-transfected cells and 2) no significant changes (>0.5-fold and <2-fold, respectively) in the ASO-NEG group compared with the mock group. Sequence searches allowing for mismatches, insertions, or deletions were performed using GGGenome (https ://GGGenome.dbcls.jp/), because BLAST software may overlook potential complementary regions.[11, 12] Finally, the results of microarray and in silico analyses were combined to identify candidate genes subject to potential off-target effects of AmNA-modified Anti-*SYT13* ASOs.

Intracellular Signaling Mediated by *SYT13*

SYT13 is a single-pass membrane protein. We therefore investigated the effects on *SYT13* expression

of five candidate ligands that promote peritoneal metastasis of gastric cancer. Recombinant human amphiregulin (262-AR-100), C-X-C motif chemokine ligand 12 (CXCL12, 350-NS-010), delta like canonical notch ligand 1 (1818-DL-050), heparin binding EGF like growth factor (HB-EGF, 259-HE-050), and jagged canonical notch ligand 1 (1277-JG-050) were obtained from R&D Systems (Minneapolis, MN, USA).[13-16] Each protein (0, 1, 10, 100, or 1000 ng/ml) was added to MKN1 cells (5000 cells per well), and the levels of *SYT13* mRNA were determined after incubation for 72 h.

We employed two array systems to investigate intracellular signaling pathways. The Human XL Oncology Array Kit (R&D Systems, Minneapolis, MN, USA) was used to determine the relative levels of 84 human cancer-related proteins expressed by ASO-transfected NUGC4 cells in the presence and absence of *SYT13* expression. Phosphorylation of 1006 unique sites among 409 proteins in these cells, representing the AKT, MAPK, NF-κB, and JAK/STAT signaling pathways, were quantified using the PTMScan Direct Multi-Pathway Enrichment Kit (Cell Signaling Technology, Danvers, MA, USA).[17] The associations of the PI3K signal transduction pathway, represented by activation of AKT, were analyzed using a Muse PI3K Activation Dual Detection Kit (Merck Millipore). Protein expression and phosphorylation were assessed using a capillary electrophoresis method a Wes automated system (ProteinSimple, San Jose, CA, USA) according to the manufacturer’s instructions. Digital imaging of western blot data was performed using Compass for SW version 4.0.0 analysis software (ProteinSimple). Antibodies used for this purpose are listed in Table S3.

Mouse Model of Peritoneal Metastasis

Experiments using animals were performed according to the ARRIVE guidelines and were approved by the Animal Research Committee of Nagoya University (approval number 31370).[2] First, we evaluated the effects of intraperitoneal administration of AmNA-modified anti-*SYT13* ASOs compared with those of siRNAs. MKN1 or NUGC4 cells (1×10^6 each) stably expressing luciferase were implanted into the abdominal cavities of BALBc$^{nu/nu}$ mice (males, 8 weeks old). Mice (n = 4, each condition) were intraperitoneally injected twice after implantation each week for 6 weeks with 500 µL of glucose, 0.2 mg (approximately 10 mg/kg) of ASO-NEG, and 0.2 mg of hSYT13-4378 or 0.2 mg of siRNA, in the presence of 15 mM $CaCl_2$. The mice were killed 6 weeks after implantation, and the total volume of peritoneal nodules was measured at laparotomy. We used an In Vivo Imaging System (IVIS) Lumina (Xenogen, Alameda, CA, USA) to noninvasively monitor the burden of peritoneal metastasis, as previously described.[10]

Next, the effects of AmNA-modified anti-*SYT13* ASOs on survival was evaluated after implantation of 2×10^6 NUGC4 cells into the abdominal cavities of BALBc$^{nu/nu}$ mice (males, 8 weeks old). Mice (n = 8, each condition) were intraperitoneally injected twice each week for 12 weeks postimplantation with 500 µL of glucose, 0.2 mg (approximately 10 mg/kg) of ASO-NEG, 0.2 mg of hSYT13-4378, or 0.2 mg of hSYT13-4733, in the presence of 15 mM $CaCl_2$. One satellite mouse was allocated from each treatment group to observe macroscopic findings 8 weeks after

implantation.

Toxicity of Intraperitoneal Administration of AmNA-modified Anti-SYT13 ASOs

To assess the effects of ASOs on a mouse model of intraperitoneal metastasis, 1.5 × 10^6 NUGC4 cells were implanted into the abdominal cavities of BALBc$^{nu/nu}$ mice (males, 8 weeks old). Mice were allocated into the two groups, which were injected once or twice each week, respectively. Mice (n = 8, each condition) were intraperitoneally injected with 500 μL of glucose, 0.2 mg (approximately 10 mg/kg) of ASO-NEG, 0.06 mg (approximately 3 mg/kg) of hSYT13-4378, 0.2 mg (approximately 10 mg/kg) of hSYT13-4378, or 0.6 mg (approximately 30 mg/kg) of hSYT13-4378, in the presence of 15 mM $CaCl_2$ for 4 weeks after implantation.

To assess the safety of intraperitoneal administration of ASOs, the appearance of the skin, food consumption, and the body weights of each group were monitored. The mice were killed 4 weeks after implantation, and data were acquired as follows: total volume of peritoneal nodules, *SYT13* mRNA levels in peritoneal nodules, blood tests, and macroscopic and pathological characteristics of the liver, kidney and brain.

We next assessed whether the liver damage caused by intraperitoneal administration of ASOs was reversible. BALBc$^{nu/nu}$ mice (males, 8 weeks old, n = 4, each condition) were intraperitoneally injected twice each week for 2 weeks after administration of 500 μL of glucose, 0.2 mg (approximately 10 mg/kg) of AmNA-modified Anti-*SYT13* ASOs (hSYT13-4378 and hSYT13-

4733), in the presence of 15 mM $CaCl_2$. Blood tests were performed 2 weeks after initiation of treatment and 2 weeks after cessation of treatment.

Statistical Analysis

The Mann–Whitney test was used to compare the differences between two groups. Kaplan–Meier curves and the log-rank test were used to analyze survival. JMP 14 software (SAS Institute Inc., Cary, NC, USA) was used for statistical analyses, and $P < 0.05$ indicates a statistically significant difference.

References

1 Kanda M, Tanaka H, Shimizu D, Miwa T, Umeda S, Tanaka C, Kobayashi D, Hattori N, Suenaga M, Hayashi M, et al. (2018). SYT7 acts as a driver of hepatic metastasis formation of gastric cancer cells. Oncogene *37*, 5355-5366.

2 Kanda M, Shimizu D, Tanaka H, Tanaka C, Kobayashi D, Hayashi M, Takami H, Niwa Y, Yamada S, Fujii T, et al. (2018). Synaptotagmin XIII expression and peritoneal metastasis in gastric cancer. Br J Surg *105*, 1349-1358.

3 Pirog EC. (2015). Immunohistochemistry and in situ hybridization for the diagnosis and classification of squamous lesions of the anogenital region. Semin Diagn Pathol *32*, 409-418.

4 Szasz AM, Lanczky A, Nagy A, Forster S, Hark K, Green JE, Boussioutas A, Busuttil R, Szabo A, Gyorffy B. (2016). Cross-validation of survival associated biomarkers in gastric cancer using transcriptomic data of 1,065 patients. Oncotarget *7*, 49322-49333.

5 Migawa MT, Shen W, Wan WB, Vasquez G, Oestergaard ME, Low A, De Hoyos CL, Gupta R, Murray S, Tanowitz M, et al. (2019). Site-specific replacement of phosphorothioate with alkyl phosphonate linkages enhances the therapeutic profile of gapmer ASOs by modulating interactions with cellular proteins. Nucleic Acids Res *47*, 5465-5479.

6 Burdick AD, Sciabola S, Mantena SR, Hollingshead BD, Stanton R, Warneke JA, Zeng M, Martsen E, Medvedev A, Makarov SS, et al. (2014). Sequence motifs associated with hepatotoxicity of locked nucleic acid--modified antisense oligonucleotides. Nucleic Acids Res *42*, 4882-4891.

7 Harada T, Matsumoto S, Hirota S, Kimura H, Fujii S, Kasahara Y, Gon H, Yoshida T, Itoh T, Haraguchi N, et al. (2019). Chemically Modified Antisense Oligonucleotide Against ARL4C Inhibits Primary and Metastatic Liver Tumor Growth. Mol Cancer Ther *18*, 602-612.

8 Ohto U, Shibata T, Tanji H, Ishida H, Krayukhina E, Uchiyama S, Miyake K, Shimizu T. (2015). Structural basis of CpG and inhibitory DNA recognition by Toll-like receptor 9. Nature *520*, 702-705.

9 Kanda M, Shimizu D, Fujii T, Sueoka S, Tanaka Y, Ezaka K, Takami H, Tanaka H, Hashimoto R, Iwata N, et al. (2016). Function and diagnostic value of Anosmin-1 in gastric cancer progression. Int J Cancer *138*, 721-730.

10 Kanda M, Shimizu D, Tanaka H, Tanaka C, Kobayashi D, Hayashi M, Iwata N, Niwa Y, Yamada S, Fujii T, et al. (2018). Significance of SYT8 For the Detection, Prediction, and Treatment of Peritoneal Metastasis From Gastric Cancer. Ann Surg *267*, 495-503.

11 Naito Y, Bono H. (2012). GGRNA: an ultrafast, transcript-oriented search engine for genes and transcripts. Nucleic Acids Res *40*, W592-596.

12 Yoshida T, Naito Y, Yasuhara H, Sasaki K, Kawaji H, Kawai J, Naito M, Okuda H, Obika S, Inoue T. (2019). Evaluation of off-target effects of gapmer antisense oligonucleotides using human cells. Genes Cells *24*, 827-835.

13 Busser B, Sancey L, Brambilla E, Coll JL, Hurbin A. (2011). The multiple roles of amphiregulin in human cancer. Biochim Biophys Acta *1816*, 119-131.

14 Huang CC, Cheng SH, Wu CH, Li WY, Wang JS, Kung ML, Chu TH, Huang ST, Feng CT, Huang SC, et al. (2019). Delta-like 1 homologue promotes tumorigenesis and epithelial-mesenchymal transition of ovarian high-grade serous carcinoma through activation of Notch signaling. Oncogene *38*, 3201-3215.

15 Izumi D, Ishimoto T, Miyake K, Sugihara H, Eto K, Sawayama H, Yasuda T, Kiyozumi Y, Kaida T, Kurashige J, et al. (2016). CXCL12/CXCR4 activation by cancer-associated fibroblasts promotes integrin beta1 clustering and invasiveness in gastric cancer. Int J Cancer *138*, 1207-1219.

16 Yasumoto K, Yamada T, Kawashima A, Wang W, Li Q, Donev IS, Tacheuchi S, Mouri H, Yamashita K, Ohtsubo K, et al. (2011). The EGFR ligands amphiregulin and heparin-binding egf-like growth factor promote peritoneal carcinomatosis in CXCR4-expressing gastric cancer. Clin Cancer Res *17*, 3619-3630.

17 Kanda M, Shimizu D, Sawaki K, Nakamura S, Umeda S, Miwa T, Tanaka H, Tanaka C, Hayashi M, Iguchi Y, et al. (2020). Therapeutic monoclonal antibody targeting of neuronal pentraxin receptor to control metastasis in gastric cancer. Mol Cancer *19*, 131.
