# Supplementary material for: Amido-Bridged Nucleic Acid-Modified Antisense Oligonucleotides Targeting SYT13 to Treat Peritoneal Metastasis of Gastric Cancer
Source: Mol Ther Nucleic Acids. 2020 Oct 6;22:791–802. doi: 10.1016/j.omtn.2020.10.001 (PMC7644579; doi:10.1016/j.omtn.2020.10.001)
Supplement: Document S2. Article plus Supplemental Information [file mmc2.pdf]

Molecular Therapy
Nucleic Acids
Original Article

Amido-Bridged Nucleic Acid-Modified Antisense Oligonucleotides Targeting *SYT13* to Treat Peritoneal Metastasis of Gastric Cancer

Mitsuro Kanda,[1,4] Yuuya Kasahara,[2,3,4] Dai Shimizu,[1] Takashi Miwa,[1] Shinichi Umeda,[1] Koichi Sawaki,[1] Shunsuke Nakamura,[1] Yasuhiro Kodera,[1] and Satoshi Obika[2,3]

[1]Department of Gastroenterological Surgery (Surgery II), Nagoya University Graduate School of Medicine, Nagoya, Japan; [2]Graduate School of Pharmaceutical Sciences, Osaka University, Osaka, Japan; [3]National Institutes of Biomedical Innovation, Health and Nutrition (NIBIOHN), Osaka, Japan

Patients with peritoneal metastasis of gastric cancer have dismal prognosis, mainly because of inefficient systemic delivery of drugs to peritoneal tumors. We aimed to develop an intraperitoneal treatment strategy using amido-bridged nucleic acid (AmNA)-modified antisense oligonucleotides (ASOs) targeting synaptotagmin XIII (*SYT13*) and to identify the function of *SYT13* in gastric cancer cells. We screened 71 candidate oligonucleotide sequences according to *SYT13*-knockdown efficacy, *in vitro* activity, and off-target effects. We evaluated the effects of *SYT13* knockdown on cellular functions and signaling pathways, as well as the effects of intraperitoneal administration to mice of AmNA-modified anti-SYT13 ASOs. We selected the ASOs (designated hSYT13-4378 and hSYT13-4733) with the highest knockdown efficiencies and lowest off-target effects and determined their abilities to inhibit cellular functions associated with the metastatic potential of gastric cancer cells. We found that *SYT13* interfered with focal adhesion kinase (FAK)-mediated intracellular signals. Intraperitoneal administration of hSYT13-4378 and hSYT13-4733 in a mouse xenograft model of metastasis inhibited the formation of peritoneal nodules and significantly increased survival. Reversible, dose- and sequence-dependent liver damage was induced by ASO treatment without causing abnormal morphological and histological changes in the brain. Intra-abdominal administration of AmNA-modified anti-SYT13 ASOs represents a promising strategy for treating peritoneal metastasis of gastric cancer.

INTRODUCTION

Peritoneal metastasis represents a devastating form of gastric cancer progression despite intensive efforts to improve the efficacy of systemic chemotherapy.[1,2] A major impediment to this strategy is that a small fraction of a drug is delivered to peritoneal tumors.[3,4] Thus, direct intraperitoneal chemotherapy represents a reasonable alternative. For example, the phase III PHOENIX-GC Trial was conducted to prove this concept.[5] Although it failed to show statistically significant superiority of intraperitoneal paclitaxel plus systemic chemotherapy, the data indicate a possible clinical benefit.[5] These findings stress the importance of developing more effective drugs for this purpose.

We recently reported that synaptotagmin XIII (*SYT13*) contributes to peritoneal metastasis of gastric cancer.[6] Thus, *SYT13* is specifically expressed in primary cancer tissues from such patients, and in a mouse model of peritoneal metastasis, intraperitoneal administration of an *SYT13*-specific small interfering RNA (siRNA) significantly inhibits the growth of peritoneal nodules and prolongs survival.[6] However, serious problems must be addressed to translate these findings to the clinic, which include limited efficacy and drug delivery using transfection techniques.

The use of antisense oligonucleotides (ASOs) that inhibit the expression of *SYT13* may provide an alternative therapeutic approach, although ASOs are vulnerable to endogenous nucleases, and their delivery to target tissues is inefficient.[7,8] Two key technologies are available to address these obstacles. First, compared with their unmodified precursors, ASOs modified by incorporating amido-bridged nucleic acids (AmNAs) with phosphorothioate-linked structures bind to mRNAs with higher affinities,[9,10] are more resistant to nucleases, and are less toxic.[11] Second, the Ca^{2+} enrichment medium (CEM) potentiates the activity of oligonucleotides, independent of net charge and structural modifications, which contributes to enhanced *in vivo* silencing activity compared with conventional transfection methods.[12]

We reasoned therefore that intraperitoneal administration of AmNA-modified anti-*SYT13* ASOs transfected using CEM represents a promising technique for treating peritoneal metastasis of gastric cancer. Here, we describe two lines of evidence that identify the function

Received 25 August 2020; accepted 1 October 2020;
https://doi.org/10.1016/j.omtn.2020.10.001.

[4]These authors contributed equally to this work.

Correspondence: Mitsuro Kanda, MD, PhD, FACS, Department of Gastroenterological Surgery (Surgery II), Nagoya University Graduate School of Medicine, 65 Tsurumai-cho, Showa-ku, Nagoya 466-8550, Japan.
E-mail: m-kanda@med.nagoya-u.ac.jp

This is an open access article under the CC BY license (http://creativecommons.org/licenses/by/4.0/).

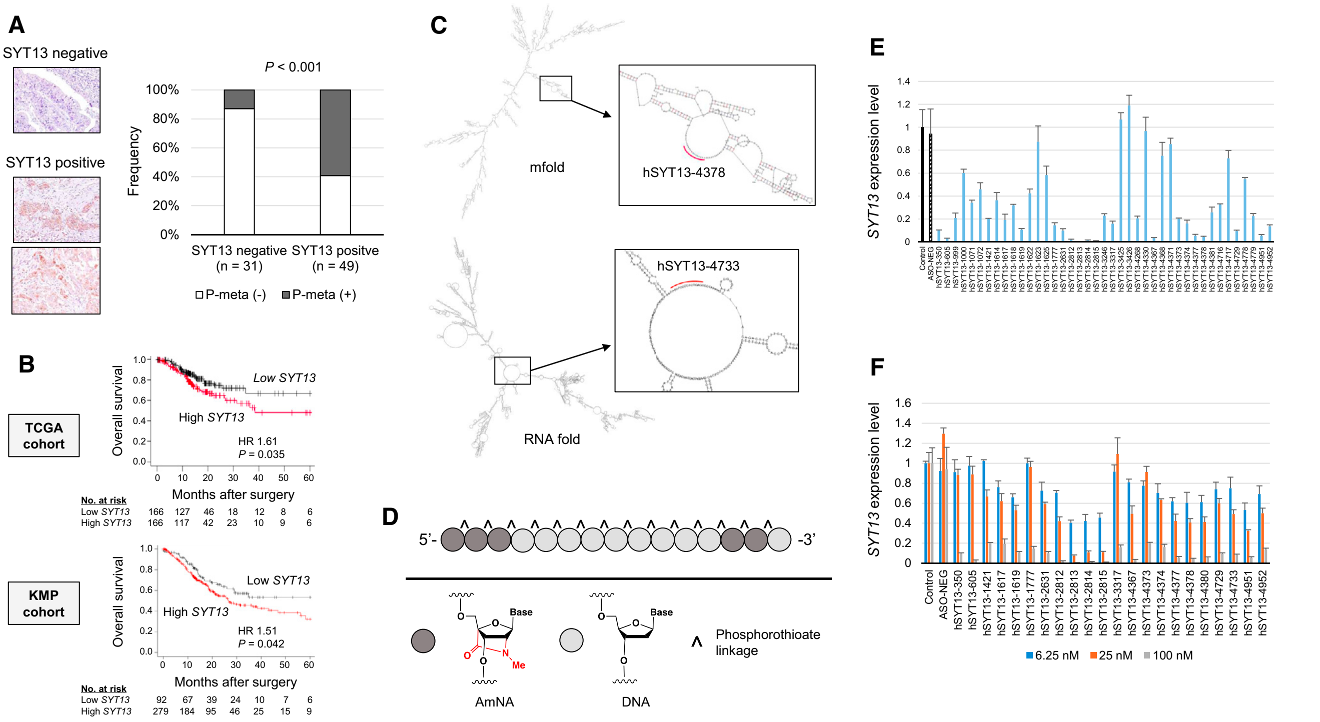

Figure 1. Clinical Relevance of *SYT13* Expression and Design of AmNA-Modified Anti-SYT13 ASOs

(A) Immunohistochemical analysis of a pathological T4a tumor. SYT13 expression at the primary cancer component was associated with peritoneal metastasis. (B) Prognosis and *SYT13* expression in TCGA and Kaplan-Meier-plotter cohorts. (C) The predicted loop structure of *SYT13* mRNA. (D) Structures of AmNA-modified anti-SYT13 ASOs. (E) Initial screening of ASOs according to knockdown efficacy of *SYT13* expression. (F) Second screening of ASOs according to concentration-dependent knockdown efficacy of *SYT13* expression. Error bars indicate the standard deviation.

of *SYT13* in gastric cancer cells and indicate that AmNA-modified, *SYT13*-specific ASOs show promise for treating peritoneal metastasis of gastric cancer.

RESULTS

Clinical Relevance of *SYT13* Expression

Representative sections with positive or negative SYT13 expression are shown in Figure 1A. Among 40 patients with a pathological T4a tumor, 26 expressed SYT13 at the primary cancer component. The incidence of concurrent or metachronous peritoneal metastasis was significantly higher in the SYT13-positive group compared with that of the SYT13-negative group (13% and 59%, respectively) (Figure 1A). In The Cancer Genome Atlas (TCGA) and Kaplan-Meier Plotter cohorts, high tissue expression of *SYT13* was significantly associated with poor prognosis after resection (Figure 1B).

Design and Screening of AmNA-Modified Anti-SYT13 ASOs

Target sites were identified according to the predicted loop structure of *SYT13* mRNA (Figure 1C). AmNA-modified anti-SYT13 ASOs included flanking regions of artificial nucleotides, and all phosphate groups were phosphorothioated (Figure 1D). We designed 71 sequences (Table S1), and to screen for optimal ASOs, we compared their abilities to inhibit *SYT13* mRNA expression in gastric cancer cell lines. For this purpose, we treated KATO-III cells, which expressed high levels of *SYT13* mRNA, with 100 nM of each ASO, and the top 22 ASOs were selected (Figure 1E). Each was used to transfect KATO-III cells. The highest concentration-dependent knockdown efficacies were achieved using hSYT13-605, hSYT13-2813, hSYT13-4367, hSYT13-4378, hSYT13-4380, hSYT13-4729, and hSYT13-4733, which were selected as candidate ASOs for further testing (Figure 1F).

Comparison of *In Vitro* Activities of Candidate ASOs

Alterations of functions associated with the metastatic potential of gastric cancer cells, such as proliferation, migration, invasiveness, and adhesion, were determined using the candidate ASO transfectants. Significant inhibition of proliferation was observed in cells transfected with hSYT13-4378, hSYT13-4729, and hSYT13-4733 (Figures 2A and S2A). Migration of KATO-III cells transfected with hSYT13-605, hSYT13-4378, and hSYT13-4733 was significantly inhibited (Figures 2B and S2B). Gastric cancer cells transfected with hSYT13-605, hSYT13-4378, hSYT13-4380, hSYT13-4729, and hSYT13-4733 were tested for invasion of Matrigel (Figures 2C and S2B). Adhesion of the ASO transfectants to five extracellular matrix proteins was significantly decreased by hSYT13-4729 and hSYT13-4733 (Figures 2D and S2C).

An overview of *in vitro* assays of seven candidate ASOs and seven gastric cancer cell lines is presented in Table S2. We selected

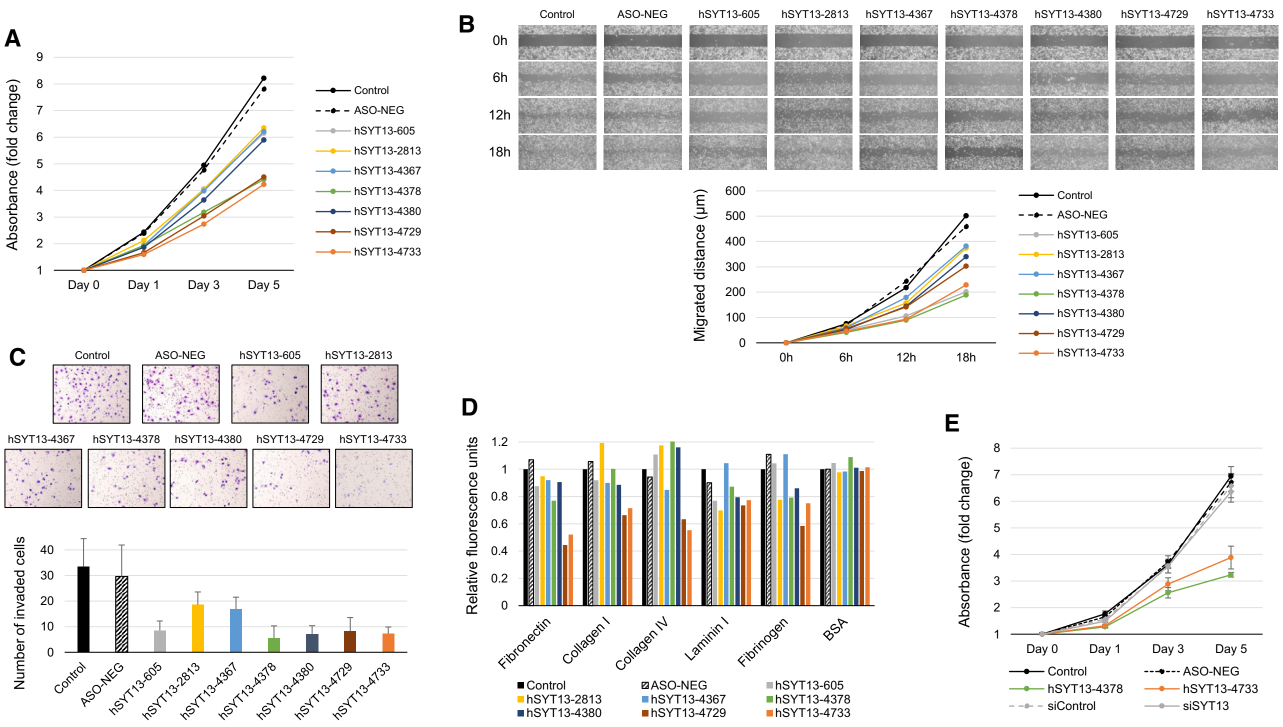

Figure 2. Effects of Candidate ASOs on Cellular Functions Associated with the Metastatic Potential of Gastric Cancer Cells

The proliferation (A), migration (B), invasiveness (C), and adhesion (D) of gastric cancer cell lines were attenuated when transfected with AmNA-modified anti-SYT13 ASOs. (E) Comparison of *in vitro* inhibitory effects of *SYT13*-specific siRNA and ASOs on proliferation of gastric cancer cells in the presence of 9 mM $CaCl_2$. $^*p < 0.05$. Error bars indicate the standard deviation.

hSYT13-4378 (15-mer) and hSYT13-4733 (17-mer) for further analyses. In contrast, the *SYT13*-specific siRNA showed little *in vitro* inhibitory effect on the proliferation of gastric cancer cells in the presence of 9 mM $CaCl_2$, in the absence of transfection agents (Figure 2E). Figures S1 and S2 show consistent results for proliferation, migration, invasion, and adhesion activities of various gastric cancer cell lines.

Influence of SYT13 Knockdown on Apoptosis, the Cell Cycle, and Cancer Stemness

To determine if *SYT13* knockdown-induced apoptosis associated with caspase activation, caspase activities were assessed. As shown in Figure 3A, ASO-mediated knockdown of *SYT13*, particularly by hSYT13-4378, increased caspase activities compared with untransfected NUGC4 cells. Further, caspase-3 and -9 activities were preferentially increased by ASO-mediated knockdown of *SYT13* expression (Figure 3A). To determine whether apoptosis induced by *SYT13* knockdown involved the mitochondrial apoptotic pathway, mitochondrial membrane potential was evaluated. The percentages of cells with loss of mitochondrial membrane potential (depolarized/live cells) were increased by ASO-mediated knockdown of *SYT13* expression, particularly by hSYT13-4378 (Figure 3B).

We used propidium iodide staining to determine the effect of SYT13 knockdown on the cell cycle. Significantly fewer NUGC4 cells transfected with hSYT13-4378 were in G1 phase compared with the controls (Figure 3C). The aldehyde dehydrogenase (ALDH) assay was used to detect the presence of subpopulations with cancer stem cell-like properties versus control cells. The percentage of NUGC4 cells expressing the stemness marker ALDH was decreased by ASO-mediated knockdown of *SYT13* expression, particularly by hSYT13-4733, compared with that of the untransfected control and ASO-NEG (ASO for negative control) cells (Figure 3D). When we employed a spheroid cell culture assay to assess cancer cell stemness, we found that hSYT13-4733 significantly inhibited spheroid formation, indicative of the stemness phenotype (Figure 3E).

Effect of Forced Expression of SYT13 on Cell Proliferation

We overexpressed *SYT13* in MKN74 cells, which expressed low levels of endogenous *SYT13* mRNA (Figure 3F). Compared with the control cells transfected with the empty plasmid (pCMV-empty control), forced expression of *SYT13* increased the proliferation in MKN74 cells by 1.5-fold after 4 days (Figure 3F).

Effects of AmNA-Modified Anti-SYT13 ASOs on Cellular Functions

To further demonstrate the effects of AmNA-modified anti-SYT13 ASOs, MKN1 cells were transfected with different concentrations of hSYT13-4378 and hSYT13-4733. Each ASO inhibited *SYT13*

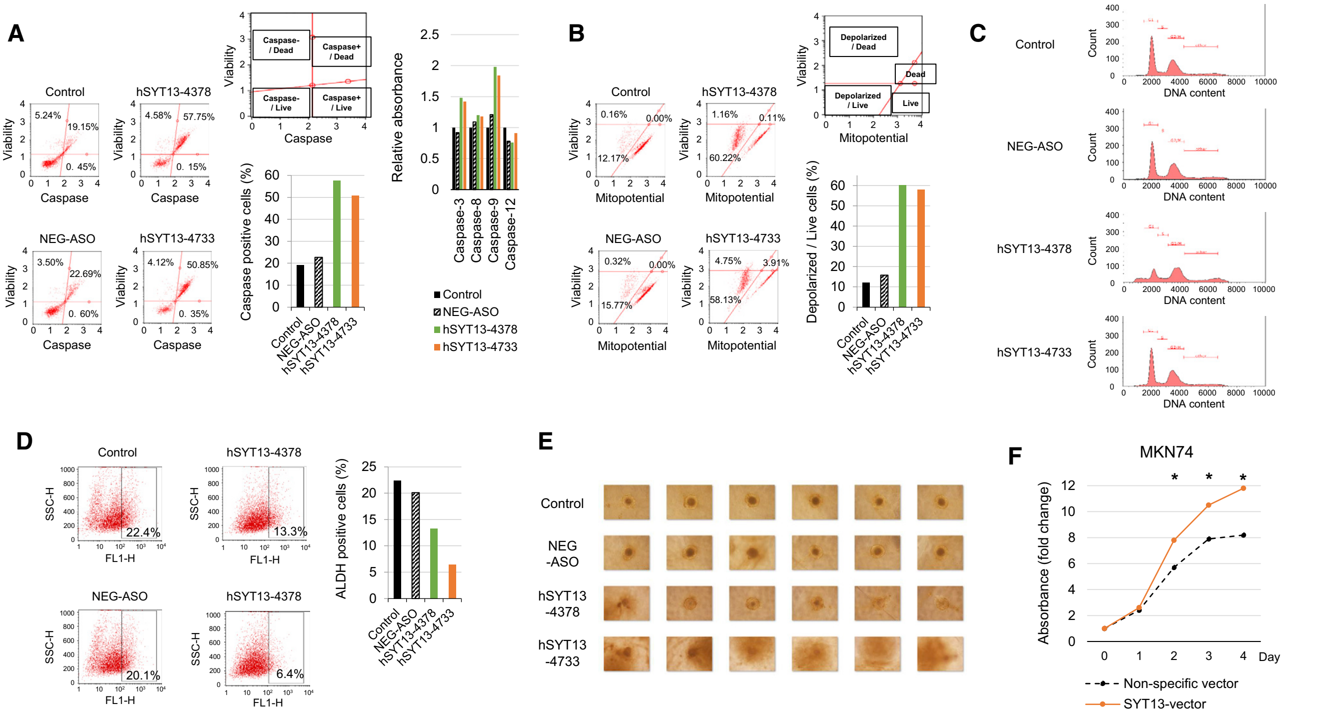

Figure 3. Influence of *SYT13* Knockdown on Apoptosis, the Cell Cycle, and Cancer Stemness

(A) Caspase activities (total or individual) were increased by *SYT13* knockdown in cells transfected with ASOs. (B) *SYT13* knockdown-induced changes in transmembrane mitochondrial potential. The distribution of cells undergoing loss of mitochondrial potential (depolarized/live) was increased by *SYT13* knockdown. (C) Cell-cycle analysis. *SYT13* knockdown decreased the proportion of cells in G1. (D) Proportions of untreated ALDH-positive cells (control, left) and after ALDH treatment (test, right). (E) Comparison of spheroid formation by MKN1 cells. (F) Forced expression of *SYT13* was confirmed using qRT-PCR. Increased proliferation compared with empty plasmid-transfected control cells (MKN74-pCMVentry). *$p < 0.05$. Error bars indicate the standard deviation.

expression (Figure 4A), as well as the proliferation (Figure 4B), invasiveness (Figure 4C), and migration (Figure 4D) of MKN1 cells. These findings were consistent when the AGS and GCIY cell lines were used (Figure S3).

Potential Off-Target Effects of AmNA-Modified Anti-SYT13 ASOs

We performed microarray analysis to detect the effects of the ASOs on candidate off-target genes, which were defined as follows: (1) $\geq$2-fold increase or $\leq$0.5-fold decrease compared with mock (untreated) cells and (2) no significant change (<0.5-fold or <2-fold) in ASO-NEG cells compared with the mock transfectants. This analysis identified 667 and 200 candidate off-target genes (including splice variants) affected by hSYT13-4378 and hSYT13-4733, respectively. An *in silico* analysis using GGGenome indicated that two identical sequences and 324 sequences with one mismatch were targeted by hSYT13-4378, and two identical sequences and five sequences with one-mismatch were targeted by hSYT13-4733.

When we combined the microarray and bioinformatics data, we found that hSYT13-4378 lacked an identical match but had two single mismatches (Jrk helix-turn-helix protein-like, transcript variant 1 and cell division cycle and apoptosis regulator 1, transcript variant 4) and 21 sequences with two mismatches. Furthermore, hSYT13-4733 lacked an identical match or one mismatch and had one sequence with two mismatches (pyruvate dehydrogenase phosphatase regulatory subunit, transcript variant X10) (Table S2). Thus, off-target effects caused by hSYT13-4378 or hSYT13-4733, particularly the latter, appeared unlikely.

Effects of SYT13 on Signal Transduction Pathways

To identify factors that potentially stimulate SYT13 expression, five candidate ligands were tested. After 72 h exposure of MKN1 cells to C-X-C motif chemokine ligand 12 (CXCL12) or heparin-binding (HB)-epidermal growth factor (EGF) SYT13, expression increased in a concentration-dependent manner (Figure 5A). To minimize the influences of off-target effects and transfection, we measured the activation of signaling proteins expressed by the hSYT13-4378, hSYT13-4733, and ASO-NEG transfectants. For this purpose, we used a Proteome Profiler antibody array and a PTMScan Direct Multi-Pathway Kit (Figure 5B). Knockdown of *SYT13* expression suppressed the phosphorylation of focal adhesion kinase (FAK)-phosphatidylinositol 3-kinase (PI3K)-AKT, out-altering that of phosphatase and tensin homolog (PTEN), and increased the phosphorylation of glycogen synthase kinase 3β (GSK3B). Moreover, downstream signaling through components of the FAK-PI3K-AKT

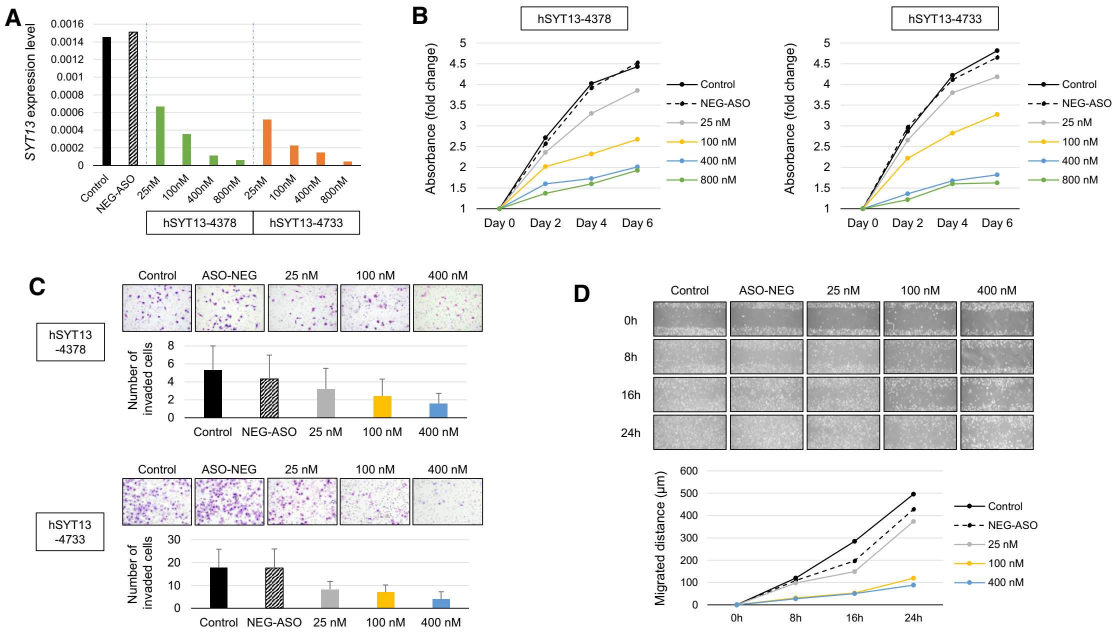

Figure 4. Concentration-Dependent Effects of hSYT13-4378 and hSYT13-4733

Concentration-dependent *in vitro* inhibitory effects of *SYT13* expression (A), cell proliferation (B), invasiveness (C), and migration (D) of MKN1 cells. *p < 0.05. Error bars indicate the standard deviation.

pathway (c-Jun N-terminal kinase [JNK], mammalian target of rapamycin [mTOR], mitogen-activated protein kinase 1 [MAPK1], and extracellular signal-regulated kinase 2 [ERK2]) was inactivated by knockdown of *SYT13*. In contrast, there was no significant influence of knockdown of *SYT13* on the phosphorylation of the JAK-STAT pathway and components of the Hippo and nuclear factor κB (NF-κB) signaling pathways. Knockdown of *SYT13* inhibited the phosphorylation of the cell-cycle regulator CDK2.

The reproducibility of findings using antibody arrays was assessed using flow cytometer-based cell analysis and digital imaging of western blots. In cells transfected with hSYT13-4378 or hSYT13-4733, the percentage of cells with unphosphorylated AKT was increased compared with mock transfected or cells transfected with ASO-NEG (Figure 5C). Digital imaging of western blots detected inhibition of the phosphorylation of FAK, AKT (Ser473), BIRC5, and CDK2, as well as activation of GSK3B in cells transfected with hSYT13-4378 or hSYT13-4733 (Figure 5D). In contrast, alterations in the phosphorylation of JAK2 and YAP were not detected (Figure 5D). Our proposed working model of the mechanism of SYT13 action in gastric cancer cells is shown in Figure 5E.

Effects of Intraperitoneal Administration of AmNA-Modified Anti-SYT13 ASOs on Intraperitoneal Metastasis

MKN1 and NUGC4 cells were used for *in vivo* experiments because they met the requirements as follows: expressed *SYT13*, a series of *in vitro* data available from ASO-mediated knockdown experiments, and formed stable xenografts in the abdominal cavity of nude mice. We used these cell lines to compare the effects of intraperitoneal administration of hSYT13-4378 (0.2 mg) with that of mock-transfected cells and cells transfected with ASO-NEG or an *SYT13*-specific siRNA. The *in vivo* imaging system (IVIS) images of mice engrafted with MKN1 cells show the luciferase signal throughout the abdominal areas in mice treated with vehicle, ASO-NEG, or siRNA groups but markedly fewer spots in mice treated with hSYT13-4378 (Figure 6A).

Macroscopic observations revealed few peritoneal nodules in the hSYT13-4378 group, 6 weeks after implantation, whereas the numbers and sizes of tumor nodules on the omentum and mesenteric tissues increased in mice treated with vehicle, ASO-NEG, and siRNA (Figure 6B). Quantitative analysis revealed inhibition of tumor growth in mice treated with hSYT13-4378 compared with those treated with vehicle, ASO-NEG, or siRNA (Figure 6B). Consistent results were observed in mice implanted with NUGC4, subjected to the treatments described above (Figure S4).

We next evaluated the therapeutic effects on survival of intraperitoneal administration of hSYT13-4378 and hSYT13-4733. Mice were intraperitoneally engrafted with NUGC4 cells and were subsequently abdominally administered vehicle, ASO-NEG, hSYT13-4378, or hSYT13-4733. The macroscopic appearances of satellite mice, 8 weeks after cell implantation, are shown in Figure 6C. Scattered peritoneal nodules were observed in engrafted mice treated with the hSYT13-4378 or hSYT13-4733. In contrast, we observed gross peritoneal metastasis across the peritoneal cavity in mice treated with vehicle or ASO-NEG (Figure 6C). Mice treated with hSYT13-4378 or

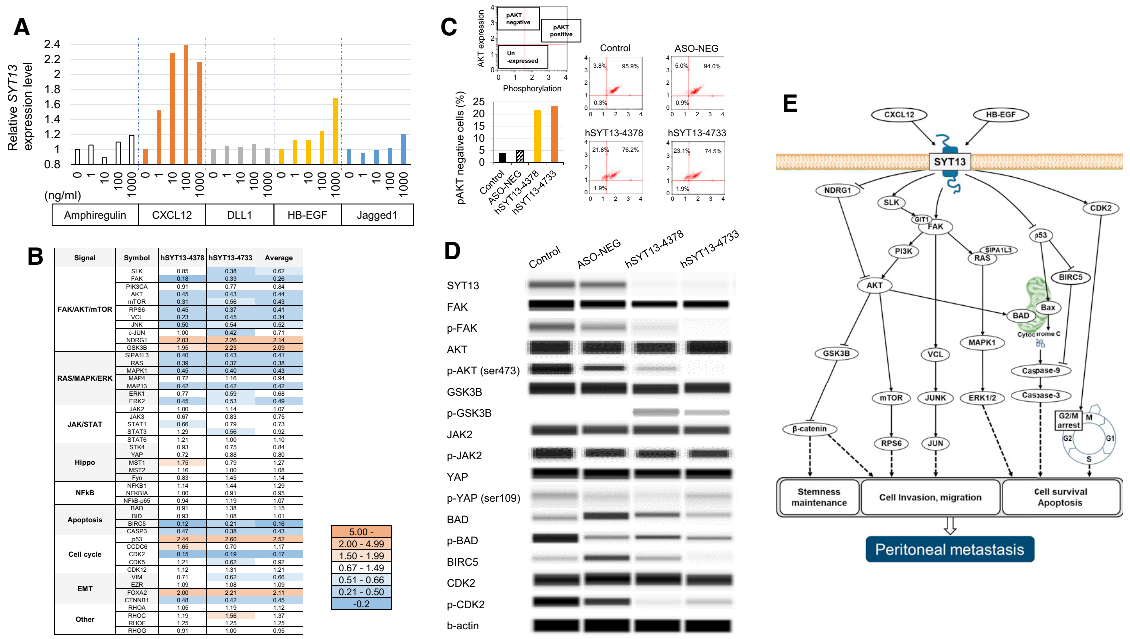

Figure 5. *SYT13* and Associated Signal Transduction Pathways

(A) *SYT13* expression levels in MKN1 cells after a 72-h exposure of five candidate ligands. (B) Overview of results acquired using two antibody systems. The fold changes for transfection of AmNA-modified anti-SYT13 ASOs are indicated by the different shades of blue. (C) Analysis of activation of AKT signaling. (D) Results of the digital western blot imaging analysis. (E) Potential signaling pathways mediated by *SYT13* in metastatic gastric cancer cells.

hSYT13-4733 survived significantly longer compared with those administered vehicle or ASO-NEG (Figure 6D).

Safety of Intraperitoneal Administration of AmNA-Modified Anti-SYT13 ASOs

The toxicity of intraperitoneal administration of hSYT13-4378 was evaluated for 4 weeks. Significant skin signs around injection sites or adhesions in the peritoneal cavity were not observed. Mice administered hSYT13-4378 exhibited loss of body weight, reduced activity, and impaired oral intake. Mice administered hSYT13-4378 lost weight and produced elevated levels of aspartate aminotransferase (AST), alanine aminotransferase (ALT), and alkaline phosphatase (ALP) (Figure 7A). Note the remarkable elevation of AST and ALT levels in mice administered 0.6 mg of hSYT13-4378 (Figure 7A). The levels of total bilirubin and hypoglycemia were significantly higher as well (Figures 7A and S5A).

Intraperitoneal administration of hSYT13-4378 did not induce detectable renal dysfunction (Figure S5A), and the brains did not show significant changes in appearance, volume, and hematoxylin and eosin staining (Figures 7B and S5B). Further, liver volumes, but not appearance of the liver, were increased (Figure 7B). Notably, hematoxylin and eosin staining revealed hepatic steatosis induced by hSYT13-4378 (Figure 7B). Macroscopic characteristics of the peritoneal cavity are shown in Figure S5C, and quantitative analysis of the total volume of peritoneal nodules revealed that the inhibitory effect of hSYT13-4378 increased in a concentration-dependent manner that did not increase with dosing intervals (Figure 7C). When we determined *SYT13* mRNA expression levels in the peritoneal nodules using qRT-PCR, we found that the intratumoral levels of *SYT13* mRNA gradually decreased with increasing concentrations of hSYT13-4378 (Figure 7D).

When we measured the levels of AST and ALT to evaluate the toxicities of hSYT13-4378 and hSYT13-4733, we did not detect elevated levels of either enzyme in mice administered hSYT13-4733, whereas their levels were transiently increased (2 weeks) in mice administered hSYT13-4378 (Figure 7E). These findings indicate that hepatotoxicity, induced by AmNA-modified anti-SYT13 ASOs, was reversible and sequence dependent. Renal dysfunction and metabolic abnormalities were undetectable in mice administered either ASO (Figure S6).

DISCUSSION

Here, we describe the development of a therapeutic strategy for inhibiting peritoneal metastasis of gastric cancer. For this purpose, we designed and synthesized ASOs targeting the mRNA encoding the transmembrane vesicular trafficking protein SYT13 that mediates synapsis and vesicle metabolism, which we previously identified as a driver of peritoneal metastasis of gastric cancer. Among a pool of 71 ASOs, we identified and focused on two with the highest efficacies, acceptable toxicities, and minimal potential off-target effects. We show here that these ASOs inhibited cellular functions associated with metastatic potential of gastric cancer cell lines in a concentration-dependent manner. Moreover, we employed a mouse tumor

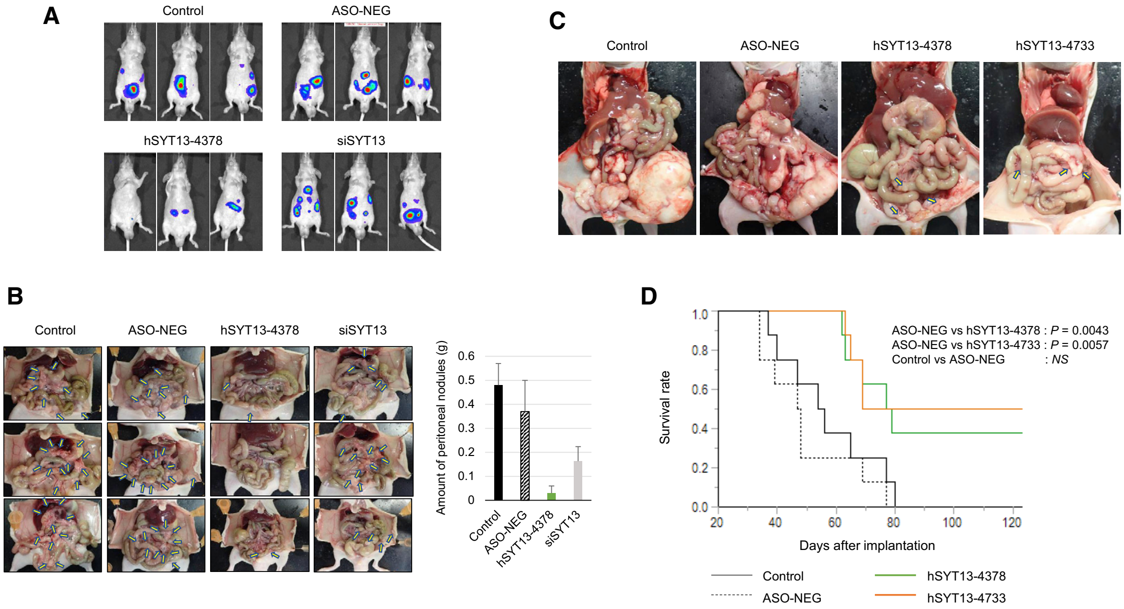

Figure 6. Therapeutic Effects of Intraperitoneal Administration of AmNA-Modified anti-SYT13 ASOs

(A) IVIS analysis of representative mice in each treatment group, 3 weeks after engraftment with MKN1 cells. (B) Macroscopic appearance of peritoneal nodules, 6 weeks after implantation of MKN1 cells, and comparison of the total volume of peritoneal nodules in each treatment group. (C) The macroscopic appearance of satellite mice 8 weeks after implantation of NUGC4 cells. (D) Survival of mice treated with intraperitoneal administration of vehicle, ASO-NEG, hSYT13-4378, or hSYT13-4733. Error bars indicate the standard deviation.

xenograft model to show that intraperitoneal administration of the ASOs inhibited the formation of peritoneal nodules and significantly prolonged survival with acceptable toxicity. Our present findings may be applicable to the treatment of other tumors with metastatic potential because SYT13 inhibits multiple intracellular proliferative signals generated by the activation of FAK.

Peritoneal metastasis is the most life-threating form of gastric cancer, and no effective systemic therapy is available.[13,14] The main reason for the unsuccessful outcomes of systemic therapy (intravenous or oral administration) is insufficient delivery of an antitumor agent to tumors scattered throughout the peritoneum.[5] In contrast, intraperitoneal chemotherapy generally exposes such tumors to high concentrations of drugs, thereby minimizing blood concentrations that induce toxicity.[15] For example, relatively large molecules, such as paclitaxel, are slowly absorbed from the peritoneum when systemically administered.[16,17] A phase III clinical trial identified certain patients with marked responses to intraperitoneal paclitaxel who experienced prolonged survival compared with patients who were administered systemic chemotherapy alone, although the difference was not statistically significant.[5] We obviously require effective therapeutic agents that can be directly administered to the peritoneum.

SYT13 binds to cellular membranes in a calcium-independent fashion to mediate the transport of biomolecules.[18–20] Our previous pattern-specific transcriptome analysis of metastasis found that *SYT13* is specifically expressed in patients with peritoneal metastasis of gastric cancer.[6] Furthermore, our present immunohistochemical (IHC) analysis of an institutional cohort and expression analysis of two external cohorts confirm our previous study, and here, we show that tissue expression of *SYT13* was significantly associated with peritoneal metastasis and poor prognosis of patients in all cohorts.[6] These results establish *SYT13* as an attractive target of chemotherapy and that measurement of *SYT13* expression shows promise as a supplemental diagnostic tool.

Moreover, we recently found that *SYT13* mRNA levels in peritoneal fluid serve as a promising approach to detect floating intraperitoneal microcells and that patients with negative cytology at gastrectomy harbor a recurrence of peritoneal metastasis.[21] Such measurements are relatively easy to perform because patients are administered intraperitoneal chemotherapy through an intra-abdominal access port, which allows repeated collection of lavage fluid at an outpatient clinic. Measurement of *SYT13* mRNA levels in peritoneal fluid may therefore serve as a new method to monitor peritoneal recurrence or response to treatment if temporal changes in molecular markers in lavage fluid are associated with therapeutic effects.

The development of peritoneal metastasis proceeds when gastric cancer cells seed the peritoneum, survive in the microenvironment of the abdominal cavity, adhere to the distant mesothelium, invade the basement membrane, and induce angiogenesis that supports tumor growth.[22] These events are not mutually exclusive and may involve multiple molecular mechanisms that operate during peritoneal

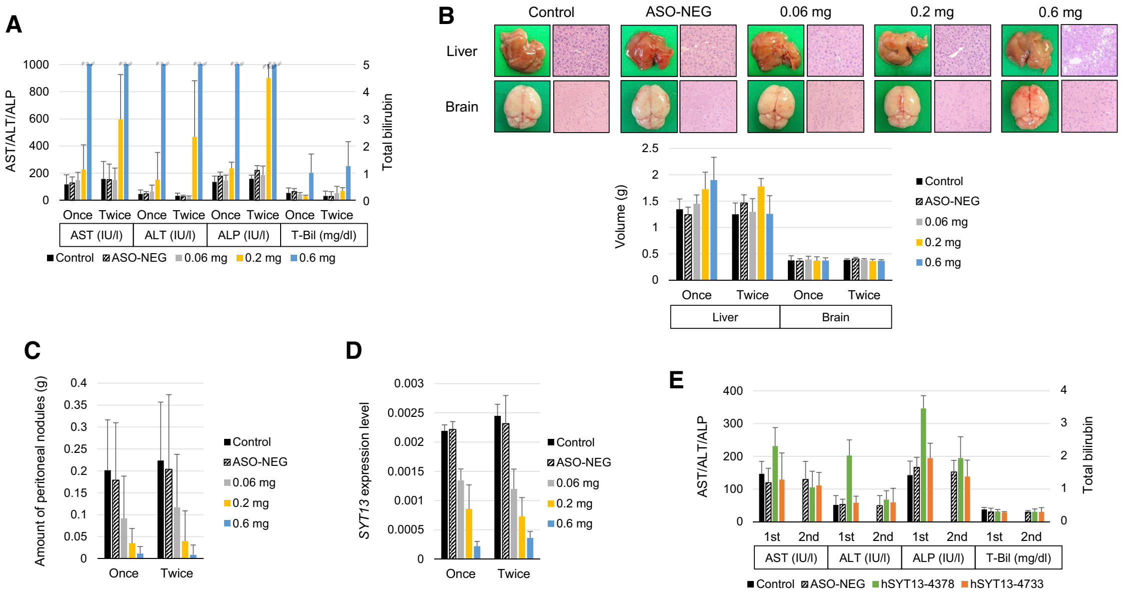

Figure 7. Toxicity of Intraperitoneal Administration of AmNA-Modified Anti-SYT13 ASOs

(A) Liver function after treatment for 4 weeks. (B) Appearance (twice weekly groups), histological findings (twice weekly groups), and volumes of the liver and brain after treatment for 4 weeks. (C) Total volume of peritoneal nodules of each treatment group. (D) *SYT13* mRNA levels in the peritoneal nodules. (E) Blood tests after 2 weeks administration of 0.2 mg hSYT13-4378 or hSYT13-4733, twice weekly and 2 weeks after treatment ceased. Error bars indicate the standard deviation.

dissemination.[4,23] Our present knockdown experiments using ASOs reveal that SYT13 controls cellular functions during each stage of seed formation, such as proliferation, invasion, migration, and adhesion of gastric cancer cells. Furthermore, these phenotypic attributes were enhanced through forced expression of *SYT13*. Moreover, knockdown of *SYT13* expression suppressed spheroid formation and stemness that mediate the formation of peritoneal metastasis through enabling cancer cells to survive in a hypoxic environment and to acquire resistance to anticancer drugs. We believe that it is reasonable to conclude that knockdown of *SYT13* expression will serve as an effective therapy for inhibiting the proliferation of metastatic gastric cancer cells that populate the peritoneum.

To understand the mechanism of action of therapeutics that inhibit *SYT13* expression, we performed a comprehensive analysis of intracellular signaling pathways. Specifically, we show that CXCL12 and HB-EGF likely stimulated SYT13 on the cell membrane. CXCL12 is a chemokine that signals through guanine nucleotide-binding proteins to initiate intracellular signaling cascades that promote migration toward the source of the chemokine. Signaling induced by CXCL12, when it binds its receptor CXCR4, contributes to the metastasis of solid cancers and cell migration via the mTOR pathway. CXCL12 is highly expressed by peritoneal mesothelial cells, and higher levels of CXCL12 are detected in malignant ascites fluid from patients with peritoneal metastasis of gastric cancer.[24,25] Further, the CXCL12/CXCR4 axis mediates cell migration.[26] For example, Izumi et al.[25] evaluated the tumor-promoting effects of CXCL12 derived from cancer-associated fibroblasts and found that inhibition of CXCL12 production decreases the invasiveness of gastric cancer cells via the suppression of integrin β1/FAK signaling. These results are consistent with our pathway analysis of *SYT13* (Figure 5E). HB-hepatocyte growth factor (HGF), which binds the EGF receptor (EGFR), is a precursor of a type I transmembrane protein that is expressed on the cell surface (pro-HB-EGF).[27] Yasumoto et al.[28] reported that HB-EGF, which is abundant in ascites fluids of patients with gastric cancer, induces the migration of fibroblasts to contribute to the formation of a microenvironment that promotes peritoneal metastasis of gastric cancer. Our data suggest that the malignant effects of SYT13 signaling are mediated by its interactions with CXCL12 and HB-HGF.

We identified important downstream signals transmitted by SYT13 that were mainly mediated by FAK. The PI3K-AKT-mTOR pathway regulates numerous cellular processes. For example, in various cancers, the PI3K-AKT-mTOR pathway is activated and frequently upregulated because of biallelic loss of PTEN, activating mutations in AKT1 and PIK3CA/B, and overexpression of certain growth factors.[29] Our results indicate potentially complex interactions between SYT13 and the PI3K-AKT-mTOR pathway, which likely contribute to enhancing the survival of gastric cancer cells.[30,31] Moreover, our pathway analysis indicates that the RAS-MAPK signal was influenced by SYT13 through regulation of FAK, which affected SYT13-mediated peritoneal metastasis.

The localization of SYT13 in the cell membrane exposes only its first five N-terminal amino acid residues to the extracellular environment.[18] Therefore, nucleic acid drugs potentially serve as a better approach to targeted therapy than therapeutic antibodies. The

strengths of our AmNA-ASOs are as follows: (1) oligonucleotide sequences with enhanced target specificity, lack of significant homology with the mouse counterpart of *SYT13*, and minimal toxicity; (2) chemical modifications extend the half-life of therapeutic ASOs to prolong exposure of tumors on the peritoneal wall;[8,32] (3) higher binding affinity to target molecules that enhance drug efficacy;[33] and (4) chemical modifications that change the lipophilicity of ASOs to confer resistance to absorption in the peritoneal cavity.[7,34] The two ASOs studied here were carefully selected from 71 sequences through multistep screening for high knockdown efficacy, effective *in vitro* activity, and potentially minimal off-target effects.

The inhibitory effect on peritoneal metastasis by intraperitoneal administration of hSYT13-4378 was exhibited using two cell lines with different phenotypes (MKN1, differentiated; NUGC4, poorly differentiated) of gastric cancer cell lines. The selected ASOs, hSYT13-4378 and hSYT13-4733, significantly prolonged the survival of significant numbers of mice (33% and 50%, respectively) and conferred long-term survival, which may be considered a cure. Furthermore, the levels of *SYT13* mRNA in peritoneal nodules were decreased in the presence of increasing concentrations of the ASOs, suggesting their stabilities in intraperitoneal cavity and ascites fluids, which enhance their incorporation into cancer cells in the peritoneum.

The most frequent and important adverse effect of ASOs is hepatotoxicity.[35] Furthermore, *SYT13* is abundantly expressed in the nervous system.[20,36] Therefore, the toxicity of intraperitoneal administration of ASOs was evaluated, paying particular attention to their effects on the liver and brain. Intraperitoneal administration of hSYT13-4378 did not cause significant skin signs around injection sites or behavioral abnormalities. Although the inhibitory effect on the formation of peritoneal metastasis and suppression of *SYT13* expression in tumor nodules was concentration dependent, liver damage was caused by $\geq$0.2 mg hSYT13-4378, and hepatic steatosis occurred at the maximum dose (0.6 mg), which debilitated the mice. No significant change was observed in appearance, volume, and histology of the brain in mice treated with hSYT13-4378. Interestingly, liver damage was not apparent when 0.2 mg of hSYT13-4733 was administered twice each week. Additionally, weekly doses of 0.2 mg of hSYT13-4378 alleviated liver damage, and the treatment effects were similar when this ASO was administered once and twice each week. Moreover, the liver damage caused by the ASOs was reversible. These are important basic data that will serve to guide future preclinical and clinical studies. With the consideration of its treatment efficacy, off-target data, and toxicity data, hSYT13-4733 represents the most promising ASO among the 71 initially screened.

ASOs used to treat various disorders are in different phases of clinical trials, and some nucleic acid drugs are approved for treating retinitis, homozygous familial hypercholesterolemia, and Duchenne muscular dystrophy.[8,11,32] However, over the past two decades, ASO-based cancer therapy has been unsuccessful despite substantial efforts directed toward developing rational oligonucleotide strategies to silence gene expression.[7,32] Vulnerability to endogenous nucleases, poor delivery to solid cancer tissues, and insufficient disease specificity of the target molecules represent serious obstacles to the clinical use of ASOs.

Our present findings may help to overcome these obstacles. For example, we show here that SYT13 is an attractive target molecule because it is specifically expressed in metastatic peritoneal gastric cancer cells.[6] Anti-SYT13 therapy is a new approach because *SYT13* significantly differs from the targets of existing drugs that mainly engage growth factor receptors or immune checkpoints. The second advantage is drug delivery. The molecular weight of paclitaxel (854 g/mol) is seven-fold lower than that of AmNA-modified anti-SYT13 ASOs, and it therefore slowly translocates into the blood through peritoneal permeation and subsequently stagnates in the abdominal cavity.[16,33] Thus, intraperitoneal administration may take advantage of the strengths of AmNA-modified anti-SYT13 ASOs.

Nevertheless, there remain serious problems associated with limited therapeutic efficacy and the requirement for using transfection reagents. In the present study, we demonstrate the inhibitory effect of AmNA-modified anti-SYT13 ASOs on peritoneal metastasis of gastric cancer *in vitro* and *in vivo* using the CEM method (simple adjustment of the $CaCl_2$ concentration of the ASO solution).[12] Further, the CEM method potentiates the activity of oligonucleotides, independent of net charge and modifications, and helps achieve *in vivo* silencing activity more consistently than conventional transfection methods.[12] These factors represent a significant advantage for clinical use because conventional *in vitro* transfection reagents cannot be universally applied.

In conclusion, the present study demonstrates the involvement of *SYT13* in the cellular functions associated with metastasis. Intraabdominal administration of AmNA-modified anti-SYT13 ASOs represents a promising strategy for treating the peritoneal metastasis of gastric cancer.

MATERIALS AND METHODS

More details are provided in the Supplemental Materials and Methods.

Clinical Significance of *SYT13* Expression

IHC analysis was performed using a rabbit polyclonal SYT13 antibody (OAAB02896; Aviva Systems Biology, San Diego, CA, USA) to analyze 40 sections from patients with pT4a gastric cancer, as previously described.[37] External validation cohorts were generated using global cohort data obtained from TCGA Research Network via the open source cBioPortal (https://www.cbioportal.org/)[38] and the Kaplan-Meier Plotter (http://kmplot.com/analysis/).[39]

Design and Synthesis of AmNA-Modified Anti-SYT13 ASOs

The loop structure of *SYT13* mRNA, to which ASOs binds with high affinity, was predicted using RNAfold (http://rna.tbi.univie.ac.at//cgi-bin/RNAWebSuite/RNAfold.cgi) and mfold (http://unafold.

rna.albany.edu/?q=mfold).[35] We designed 71 sequences of different lengths, considering sequence identities with *Mus musculus SYT13* mRNA and avoiding hepatotoxicity-related sequences. AmNA-modified ASOs were synthesized and purified by Gene Design, using an automated DNA synthesizer (Osaka, Japan).

In Vitro Transfection of ASOs and siRNA

Transfection of ASOs or siRNA into gastric cancer cell lines was performed using the CEM method. Cells were cultured in a 24-well plate (5,000 cells per well) and transiently transfected the next day with AmNA-modified ASOs (6.25–400 nM) or siRNAs specific for *SYT13* (100 nM [A-014082-13 and A-014082-14]) (Accell siRNA; GE Healthcare Dharmacon, Lafayette, CO, USA) in the presence of 9 mM $CaCl_2$.

Assays of Cell Function

We used a Cell Counting Kit-8 (Dojindo Molecular Technologies, Kumamoto, Japan) for cell proliferation, a wound-healing assay for cell migration, BioCoat Matrigel invasion chambers (BD Biosciences, Bedford, MA, USA) for cell invasiveness, and the CytoSelect 48-Well Cell Adhesion Assay (Cell Biolabs, San Diego, CA, USA) for cell adhesion to extracellular matrix components. These assays were performed as previously described.[2,40] To evaluate total caspase activity, a Muse MultiCaspase Kit (Merck Millipore, Billerica, MA, USA) was used. The activities of caspase-3, -8, -9, and -12 were measured using the Caspase Colorimetric Assay Kit (BioVision, Milpitas, CA, USA). Mitochondrial membrane potential and cell-cycle distribution were assessed using a Muse MitoPotential Kit (Merck Millipore) and a Muse Cell Cycle Kit (Merck Millipore), respectively. ALDH, a surrogate marker of stem/progenitor cells, was estimated using the ALDEFLOUR fluorescent reagent system (STEMCELL Technologies, Vancouver, BC, Canada). ALDH-positive cells were determined using a FACSCalibur system (BD Biosciences, Franklin Lakes, NJ, USA). The three-dimensional spheroid cultures were analyzed using PrimeSurface96U multiwell plates (Sumitomo Bakelite, Tokyo, Japan).

SYT13 Expression Vector

SYT13 cDNAs were ligated to the pFN21A HaloTag CMV Flexi Vector (Promega, Madison, WI, USA). The *SYT13* vector (0.2 mg) was used to transfect MKN74 cells (1×10^5) using the NEON Transfection System (Thermo Fisher Scientific, Waltham, MA, USA).

Microarray and *In Silico* Analysis of Candidate Genes for Potential Off-Target Effects of AmNA-Modified Anti-SYT13 ASOs

Total RNAs extracted from NUGC4 cells transfected with mock, ASO-NEG, or AmNA-modified anti-SYT13 ASOs (hSYT13-4378 and hSYT13-4733) were subjected to microarray analysis using the 3D-Gene microarray (Toray, Tokyo, Japan). Sequence searches allowing for mismatches, insertions, or deletions were performed using GGGenome (https://GGGenome.dbcls.jp/). Finally, the results of microarray and *in silico* analyses were combined to identify candidate genes subject to potential off-target effects of AmNA-modified anti-*SYT13* ASOs.

Intracellular Signaling Mediated by SYT13

Recombinant human amphiregulin (262-AR-100), CXCL12 (350-NS-010), delta-like canonical notch ligand 1 (1818-DL-050), HB-EGF-like growth factor (259-HE-050), and jagged canonical notch ligand 1 (1277-JG-050) were obtained from R&D Systems (Minneapolis, MN, USA) as candidate ligands of SYT13.[25,28,41,42] Each protein (0, 1, 10, 100, or 1,000 ng/mL) was added to MKN1 cells (5,000 cells per well), and the levels of *SYT13* mRNA were determined after incubation for 72 h.

The Human XL Oncology Array Kit (R&D Systems, Minneapolis, MN, USA) was used to determine the relative levels of 84 human cancer-related proteins expressed by ASO-transfected NUGC4 cells in the presence and absence of *SYT13* expression. Phosphorylation of 1,006 unique sites among 409 proteins in these cells was quantified using the PTMScan Direct Multi-Pathway Enrichment Kit (Cell Signaling Technology, Danvers, MA, USA).[43] Protein expression and phosphorylation were assessed using a capillary electrophoresis method with a Wes automated system (ProteinSimple, San Jose, CA, USA), according to the manufacturer's instructions. Antibodies used for this purpose are listed in Table S3.

Mouse Model of Peritoneal Metastasis

Experiments using animals were performed according to the Animal Research: Reporting of *In Vivo* Experiments (ARRIVE) guidelines and were approved by the Animal Research Committee of Nagoya University (approval number 31370).[6] First, we evaluated the effects of intraperitoneal administration of AmNA-modified anti-*SYT13* ASOs compared with those of siRNAs. MKN1 or NUGC4 cells (1×10^6 each), stably expressing luciferase, were implanted into the abdominal cavities of BALB/$c^{nu/nu}$ mice (males, 8 weeks old). Mice (n = 4, each condition) were intraperitoneally injected twice after implantation each week for 6 weeks with 500 μL of glucose, 0.2 mg (approximately 10 mg/kg) of ASO-NEG, and 0.2 mg of hSYT13-4378 or 0.2 mg of siRNA in the presence of 15 mM $CaCl_2$. We used an IVIS Lumina (Xenogen, Alameda, CA, USA) to noninvasively monitor the burden of peritoneal metastasis, as previously described.[2]

Next, the effects of AmNA-modified anti-*SYT13* ASOs on survival were evaluated after implantation of 2×10^6 NUGC4 cells into the abdominal cavities of BALB/$c^{nu/nu}$ mice (males, 8 weeks old). Mice (n = 8, each condition) were intraperitoneally injected twice each week for 12 weeks postimplantation with 500 μL of glucose, 0.2 mg (approximately 10 mg/kg) of ASO-NEG, 0.2 mg of hSYT13-4378, or 0.2 mg of hSYT13-4733 in the presence of 15 mM $CaCl_2$.

Toxicity of Intraperitoneal Administration of AmNA-Modified Anti-SYT13 ASOs

To assess the effects of ASOs on a mouse model of intraperitoneal metastasis, 1.5×10^6 NUGC4 cells were implanted into the abdominal cavities of BALB/$c^{nu/nu}$ mice (males, 8 weeks old). Mice were allocated into the two groups, which were injected once or twice each week, respectively. Mice (n = 8, each condition) were intraperitoneally injected with 500 μL of glucose, 0.2 mg (approximately 10 mg/kg) of

ASO-NEG, 0.06 mg (approximately 3 mg/kg) of hSYT13-4378, 0.2 mg (approximately 10 mg/kg) of hSYT13-4378, or 0.6 mg (approximately 30 mg/kg) of hSYT13-4378 in the presence of 15 mM $CaCl_2$ for 4 weeks after implantation.

We next assessed whether the liver damage caused by intraperitoneal administration of ASOs was reversible. BALB/$c^{nu/nu}$ mice (males, 8 weeks old; n = 4, each condition) were intraperitoneally injected twice each week for 2 weeks after administration of 500 μL of glucose and 0.2 mg (approximately 10 mg/kg) of AmNA-modified anti-*SYT13* ASOs (hSYT13-4378 and hSYT13-4733) in the presence of 15 mM $CaCl_2$. Blood tests were performed 2 weeks after initiation of treatment and 2 weeks after cessation of treatment.

Statistical Analysis

The Mann-Whitney test was used to compare the differences between two groups. Kaplan-Meier curves and the log-rank test were used to analyze survival. JMP 14 software (SAS Institute, Cary, NC, USA) was used for statistical analyses, and $p < 0.05$ indicates a statistically significant difference.

SUPPLEMENTAL INFORMATION

Supplemental Information can be found online at https://doi.org/10.1016/j.omtn.2020.10.001.

AUTHOR CONTRIBUTIONS

M.K., Y. Kasahara, and S.O. contributed to the conception and design of the study. D.S. contributed to the statistical analysis and data interpretation. T.M., S.U., K.S., and S.N. contributed to data acquisition. Y. Kodera and S.O. reviewed and revised the manuscript.

CONFLICTS OF INTEREST

The authors declare no competing interests.

ACKNOWLEDGMENTS

This work was supported by the Japan Agency for Medical Research and Development (DNW-16012 and 20lm0203132h0001).

REFERENCES

1. Shen, L., Shan, Y.S., Hu, H.M., Price, T.J., Sirohi, B., Yeh, K.H., Yang, Y.H., Sano, T., Yang, H.K., Zhang, X., et al. (2013). Management of gastric cancer in Asia: resource-stratified guidelines. Lancet Oncol. *14*, e535–e547.

2. Kanda, M., Shimizu, D., Tanaka, H., Tanaka, C., Kobayashi, D., Hayashi, M., Iwata, N., Niwa, Y., Yamada, S., Fujii, T., et al. (2018). Significance of SYT8 For the Detection, Prediction, and Treatment of Peritoneal Metastasis From Gastric Cancer. Ann. Surg. *267*, 495–503.

3. Van Cutsem, E., Sagaert, X., Topal, B., Haustermans, K., and Prenen, H. (2016). Gastric cancer. Lancet *388*, 2654–2664.

4. Wadhwa, R., Song, S., Lee, J.S., Yao, Y., Wei, Q., and Ajani, J.A. (2013). Gastric cancer-molecular and clinical dimensions. Nat. Rev. Clin. Oncol. *10*, 643–655.

5. Ishigami, H., Fujiwara, Y., Fukushima, R., Nashimoto, A., Yabusaki, H., Imano, M., Imamoto, H., Kodera, Y., Uenosono, Y., Amagai, K., et al. (2018). Phase III Trial Comparing Intraperitoneal and Intravenous Paclitaxel Plus S-1 Versus Cisplatin Plus S-1 in Patients With Gastric Cancer With Peritoneal Metastasis: PHOENIX-GC Trial. J. Clin. Oncol. *36*, 1922–1929.

6. Kanda, M., Shimizu, D., Tanaka, H., Tanaka, C., Kobayashi, D., Hayashi, M., Takami, H., Niwa, Y., Yamada, S., Fujii, T., et al. (2018). Synaptotagmin XIII expression and peritoneal metastasis in gastric cancer. Br. J. Surg. *105*, 1349–1358.

7. Castanotto, D., and Stein, C.A. (2014). Antisense oligonucleotides in cancer. Curr. Opin. Oncol. *26*, 584–589.

8. Ge, J.H., Zhu, J.W., Fu, H.Y., Shi, W.B., and Zhang, C.L. (2019). An Antisense Oligonucleotide Drug Targeting miR-21 Induces H1650 Apoptosis and Caspase Activation. Technol. Cancer Res. Treat. *18*, 1533033819892263.

9. Yahara, A., Shrestha, A.R., Yamamoto, T., Hari, Y., Osawa, T., Yamaguchi, M., Nishida, M., Kodama, T., and Obika, S. (2012). Amido-bridged nucleic acids (AmNAs): synthesis, duplex stability, nuclease resistance, and in vitro antisense potency. ChemBioChem *13*, 2513–2516.

10. Yamamoto, T., Yahara, A., Waki, R., Yasuhara, H., Wada, F., Harada-Shiba, M., and Obika, S. (2015). Amido-bridged nucleic acids with small hydrophobic residues enhance hepatic tropism of antisense oligonucleotides in vivo. Org. Biomol. Chem. *13*, 3757–3765.

11. Setoguchi, K., Cui, L., Hachisuka, N., Obchoei, S., Shinkai, K., Hyodo, F., Kato, K., Wada, F., Yamamoto, T., Harada-Shiba, M., et al. (2017). Antisense Oligonucleotides Targeting Y-Box Binding Protein-1 Inhibit Tumor Angiogenesis by Downregulating Bcl-xL-VEGFR2/-Tie Axes. Mol. Ther. Nucleic Acids *9*, 170–181.

12. Hori, S., Yamamoto, T., Waki, R., Wada, S., Wada, F., Noda, M., and Obika, S. (2015). Ca2+ enrichment in culture medium potentiates effect of oligonucleotides. Nucleic Acids Res. *43*, e128.

13. Kobayashi, D., Ishigami, H., Kanda, M., Tanaka, C., Yamaguchi, H., Kitayama, J., and Kodera, Y. (2020). Phase I Study of Intraperitoneal Administration of Paclitaxel Combined with S-1 Plus Cisplatin for Gastric Cancer with Peritoneal Metastasis. Oncology *98*, 48–52.

14. Koizumi, W., Narahara, H., Hara, T., Takagane, A., Akiya, T., Takagi, M., Miyashita, K., Nishizaki, T., Kobayashi, O., Takiyama, W., et al. (2008). S-1 plus cisplatin versus S-1 alone for first-line treatment of advanced gastric cancer (SPIRITS trial): a phase III trial. Lancet Oncol. *9*, 215–221.

15. Narahara, H., Fujitani, K., Takiuchi, H., Sugimoto, N., Inoue, K., Uedo, N., Tsukuma, H., Tsujinaka, T., Furukawa, H., and Taguchi, T. (2008). Phase II study of a combination of S-1 and paclitaxel in patients with unresectable or metastatic gastric cancer. Oncology *74*, 37–41.

16. Ishigami, H., Kitayama, J., Otani, K., Kamei, T., Soma, D., Miyato, H., Yamashita, H., Hidemura, A., Kaisaki, S., and Nagawa, H. (2009). Phase I pharmacokinetic study of weekly intravenous and intraperitoneal paclitaxel combined with S-1 for advanced gastric cancer. Oncology *76*, 311–314.

17. Ishigami, H., Kitayama, J., Kaisaki, S., Hidemura, A., Kato, M., Otani, K., Kamei, T., Soma, D., Miyato, H., Yamashita, H., and Nagawa, H. (2010). Phase II study of weekly intravenous and intraperitoneal paclitaxel combined with S-1 for advanced gastric cancer with peritoneal metastasis. Ann. Oncol. *21*, 67–70.

18. von Poser, C., and Südhof, T.C. (2001). Synaptotagmin 13: structure and expression of a novel synaptotagmin. Eur. J. Cell Biol. *80*, 41–47.

19. Jahn, J.E., Best, D.H., and Coleman, W.B. (2010). Exogenous expression of synaptotagmin XIII suppresses the neoplastic phenotype of a rat liver tumor cell line through molecular pathways related to mesenchymal to epithelial transition. Exp. Mol. Pathol. *89*, 209–216.

20. Nizzardo, M., Taiana, M., Rizzo, F., Aguila Benitez, J., Nijssen, J., Allodi, I., Melzi, V., Bresolin, N., Comi, G.P., Hedlund, E., and Corti, S. (2020). Synaptotagmin 13 is neuroprotective across motor neuron diseases. Acta Neuropathol. *139*, 837–853.

21. Nakanishi, K., Kanda, M., Umeda, S., Tanaka, C., Kobayashi, D., Hayashi, M., Yamada, S., and Kodera, Y. (2019). The levels of SYT13 and CEA mRNAs in peritoneal lavages predict the peritoneal recurrence of gastric cancer. Gastric Cancer *22*, 1143–1152.

22. Kanda, M., and Kodera, Y. (2016). Molecular mechanisms of peritoneal dissemination in gastric cancer. World J. Gastroenterol. *22*, 6829–6840.

23. Yasui, W., Sentani, K., Sakamoto, N., Anami, K., Naito, Y., and Oue, N. (2011). Molecular pathology of gastric cancer: research and practice. Pathol. Res. Pract. *207*, 608–612.

24. Domanska, U.M., Kruizinga, R.C., Nagengast, W.B., Timmer-Bosscha, H., Huls, G., de Vries, E.G., and Walenkamp, A.M. (2013). A review on CXCR4/CXCL12 axis in oncology: no place to hide. Eur. J. Cancer *49*, 219–230.

25. Izumi, D., Ishimoto, T., Miyake, K., Sugihara, H., Eto, K., Sawayama, H., Yasuda, T., Kiyozumi, Y., Kaida, T., Kurashige, J., et al. (2016). CXCL12/CXCR4 activation by cancer-associated fibroblasts promotes integrin β1 clustering and invasiveness in gastric cancer. Int. J. Cancer *138*, 1207–1219.

26. Duda, D.G., Kozin, S.V., Kirkpatrick, N.D., Xu, L., Fukumura, D., and Jain, R.K. (2011). CXCL12 (SDF1alpha)-CXCR4/CXCR7 pathway inhibition: an emerging sensitizer for anticancer therapies? Clin. Cancer Res. *17*, 2074–2080.

27. Moosavi, F., Giovannetti, E., Saso, L., and Firuzi, O. (2019). HGF/MET pathway aberrations as diagnostic, prognostic, and predictive biomarkers in human cancers. Crit. Rev. Clin. Lab. Sci. *56*, 533–566.

28. Yasumoto, K., Yamada, T., Kawashima, A., Wang, W., Li, Q., Donev, I.S., Tacheuchi, S., Mouri, H., Yamashita, K., Ohtsubo, K., and Yano, S. (2011). The EGFR ligands amphiregulin and heparin-binding egf-like growth factor promote peritoneal carcinomatosis in CXCR4-expressing gastric cancer. Clin. Cancer Res. *17*, 3619–3630.

29. Davidson, L., Maccario, H., Perera, N.M., Yang, X., Spinelli, L., Tibarewal, P., Glancy, B., Gray, A., Weijer, C.J., Downes, C.P., and Leslie, N.R. (2010). Suppression of cellular proliferation and invasion by the concerted lipid and protein phosphatase activities of PTEN. Oncogene *29*, 687–697.

30. Cai, C., Dang, W., Liu, S., Huang, L., Li, Y., Li, G., Yan, S., Jiang, C., Song, X., Hu, Y., and Gu, J. (2020). Anthrax toxin receptor 1/tumor endothelial marker 8 promotes gastric cancer progression through activation of the PI3K/AKT/mTOR signaling pathway. Cancer Sci. *111*, 1132–1145.

31. Jung, E.J., Suh, J.H., Kim, W.H., and Kim, H.S. (2020). Clinical significance of PI3K/Akt/mTOR signaling in gastric carcinoma. Int. J. Clin. Exp. Pathol. *13*, 995–1007.

32. Shimojo, M., Kasahara, Y., Inoue, M., Tsunoda, S.I., Shudo, Y., Kurata, T., and Obika, S. (2019). A gapmer antisense oligonucleotide targeting SRRM4 is a novel therapeutic medicine for lung cancer. Sci. Rep. *9*, 7618.

33. Uehara, T., Choong, C.J., Nakamori, M., Hayakawa, H., Nishiyama, K., Kasahara, Y., Baba, K., Nagata, T., Yokota, T., Tsuda, H., et al. (2019). Amido-bridged nucleic acid (AmNA)-modified antisense oligonucleotides targeting α-synuclein as a novel therapy for Parkinson's disease. Sci. Rep. *9*, 7567.

34. Harada, T., Matsumoto, S., Hirota, S., Kimura, H., Fujii, S., Kasahara, Y., Gon, H., Yoshida, T., Itoh, T., Haraguchi, N., et al. (2019). Chemically Modified Antisense Oligonucleotide Against ARL4C Inhibits Primary and Metastatic Liver Tumor Growth. Mol. Cancer Ther. *18*, 602–612.

35. Migawa, M.T., Shen, W., Wan, W.B., Vasquez, G., Oestergaard, M.E., Low, A., De Hoyos, C.L., Gupta, R., Murray, S., Tanowitz, M., et al. (2019). Site-specific replacement of phosphorothioate with alkyl phosphonate linkages enhances the therapeutic profile of gapmer ASOs by modulating interactions with cellular proteins. Nucleic Acids Res. *47*, 5465–5479.

36. Zhang, L., Fan, B., Zheng, Y., Lou, Y., Cui, Y., Wang, K., Zhang, T., and Tan, X. (2020). Identification SYT13 as a novel biomarker in lung adenocarcinoma. J. Cell. Biochem. *121*, 963–973.

37. Kanda, M., Tanaka, H., Shimizu, D., Miwa, T., Umeda, S., Tanaka, C., Kobayashi, D., Hattori, N., Suenaga, M., Hayashi, M., et al. (2018). SYT7 acts as a driver of hepatic metastasis formation of gastric cancer cells. Oncogene *37*, 5355–5366.

38. Jaiswal, P.K., Koul, S., Palanisamy, N., and Koul, H.K. (2019). Eukaryotic Translation Initiation Factor 4 Gamma 1 (EIF4G1): a target for cancer therapeutic intervention? Cancer Cell Int. *19*, 224.

39. Szász, A.M., Lánczky, A., Nagy, Á., Förster, S., Hark, K., Green, J.E., Boussioutas, A., Busuttil, R., Szabó, A., and Győrffy, B. (2016). Cross-validation of survival associated biomarkers in gastric cancer using transcriptomic data of 1,065 patients. Oncotarget *7*, 49322–49333.

40. Kanda, M., Shimizu, D., Fujii, T., Sueoka, S., Tanaka, Y., Ezaka, K., Takami, H., Tanaka, H., Hashimoto, R., Iwata, N., et al. (2016). Function and diagnostic value of Anosmin-1 in gastric cancer progression. Int. J. Cancer *138*, 721–730.

41. Busser, B., Sancey, L., Brambilla, E., Coll, J.L., and Hurbin, A. (2011). The multiple roles of amphiregulin in human cancer. Biochim. Biophys. Acta *1816*, 119–131.

42. Huang, C.C., Cheng, S.H., Wu, C.H., Li, W.Y., Wang, J.S., Kung, M.L., Chu, T.H., Huang, S.T., Feng, C.T., Huang, S.C., and Tai, M.H. (2019). Delta-like 1 homologue promotes tumorigenesis and epithelial-mesenchymal transition of ovarian high-grade serous carcinoma through activation of Notch signaling. Oncogene *38*, 3201–3215.

43. Kanda, M., Shimizu, D., Sawaki, K., Nakamura, S., Umeda, S., Miwa, T., Tanaka, H., Tanaka, C., Hayashi, M., Iguchi, Y., et al. (2020). Therapeutic monoclonal antibody targeting of neuronal pentraxin receptor to control metastasis in gastric cancer. Mol. Cancer *19*, 131.

OMTN, Volume *22*

Supplemental Information

Amido-Bridged Nucleic Acid-Modified Antisense Oligonucleotides Targeting *SYT13* to Treat Peritoneal Metastasis of Gastric Cancer

Mitsuro Kanda, Yuuya Kasahara, Dai Shimizu, Takashi Miwa, Shinichi Umeda, Koichi Sawaki, Shunsuke Nakamura, Yasuhiro Kodera, and Satoshi Obika

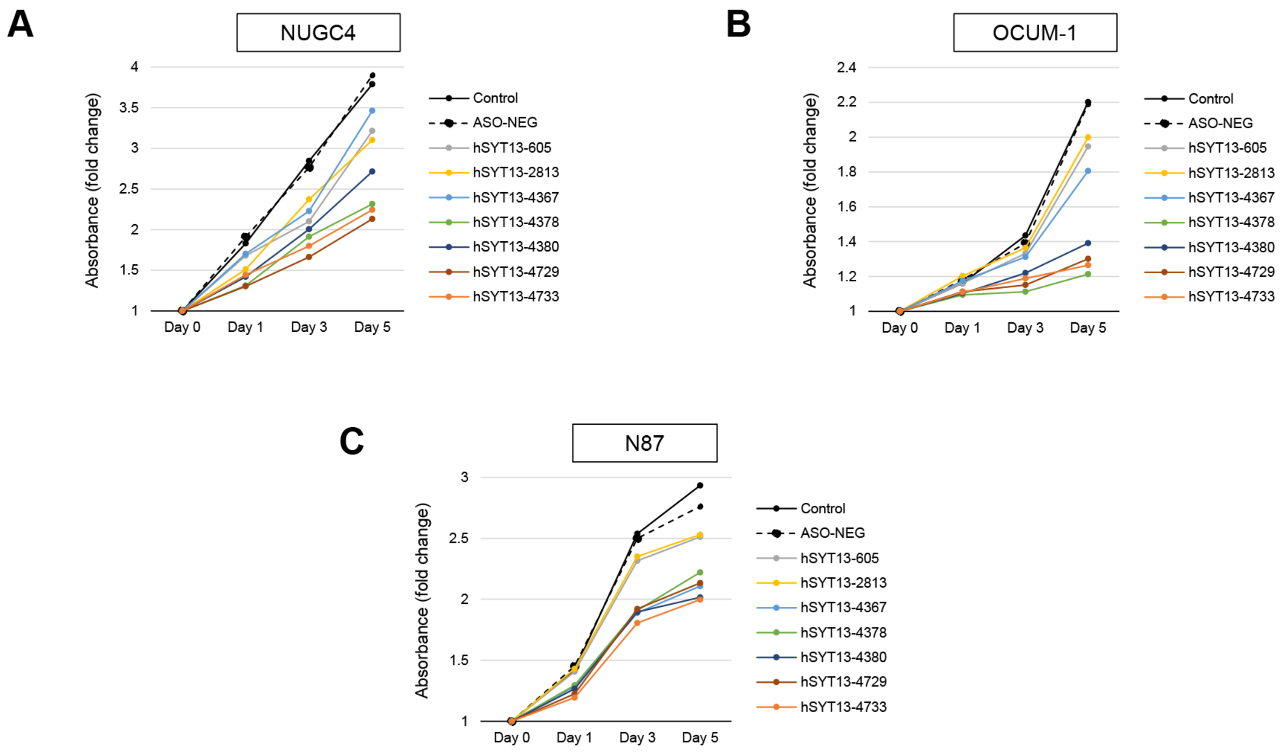

Figure S1. Comparison of effects of ASOs on the proliferation of the gastric cancer cell lines NUGC4, OCUM-1, and N87.

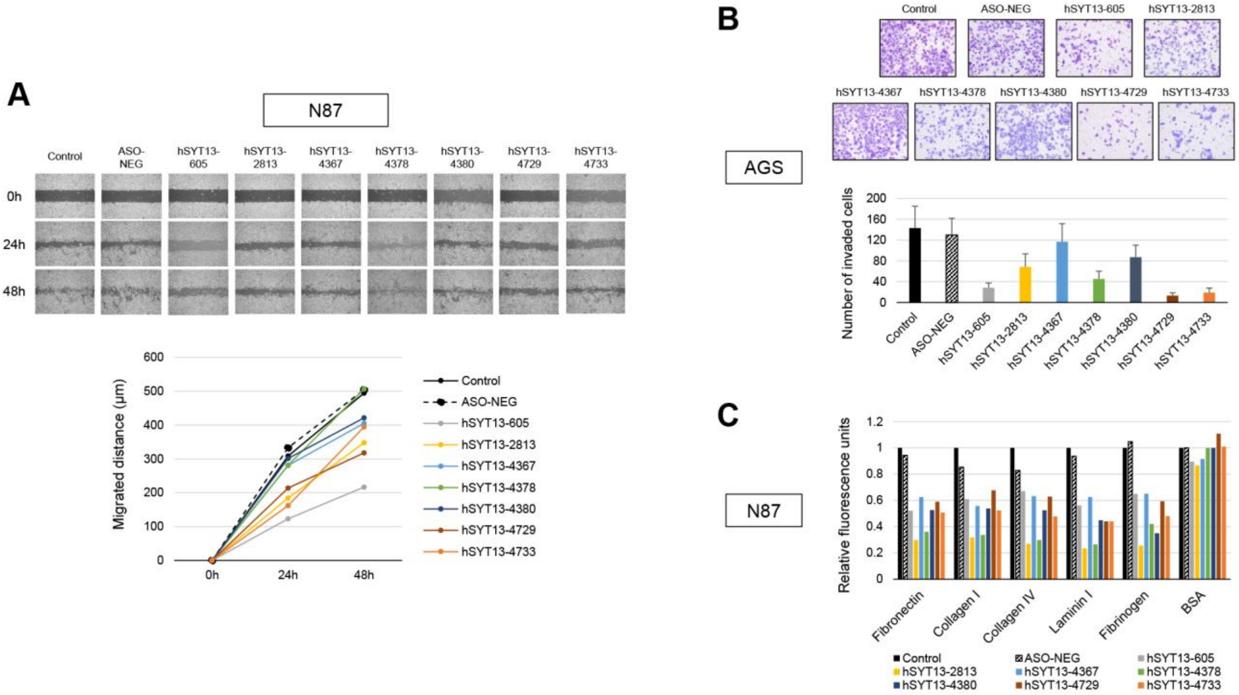

Figure S2. Comparison of effects of ASOs on the migration (*A*), invasiveness (*B*), and adhesion (*C*) of gastric cancer cells.

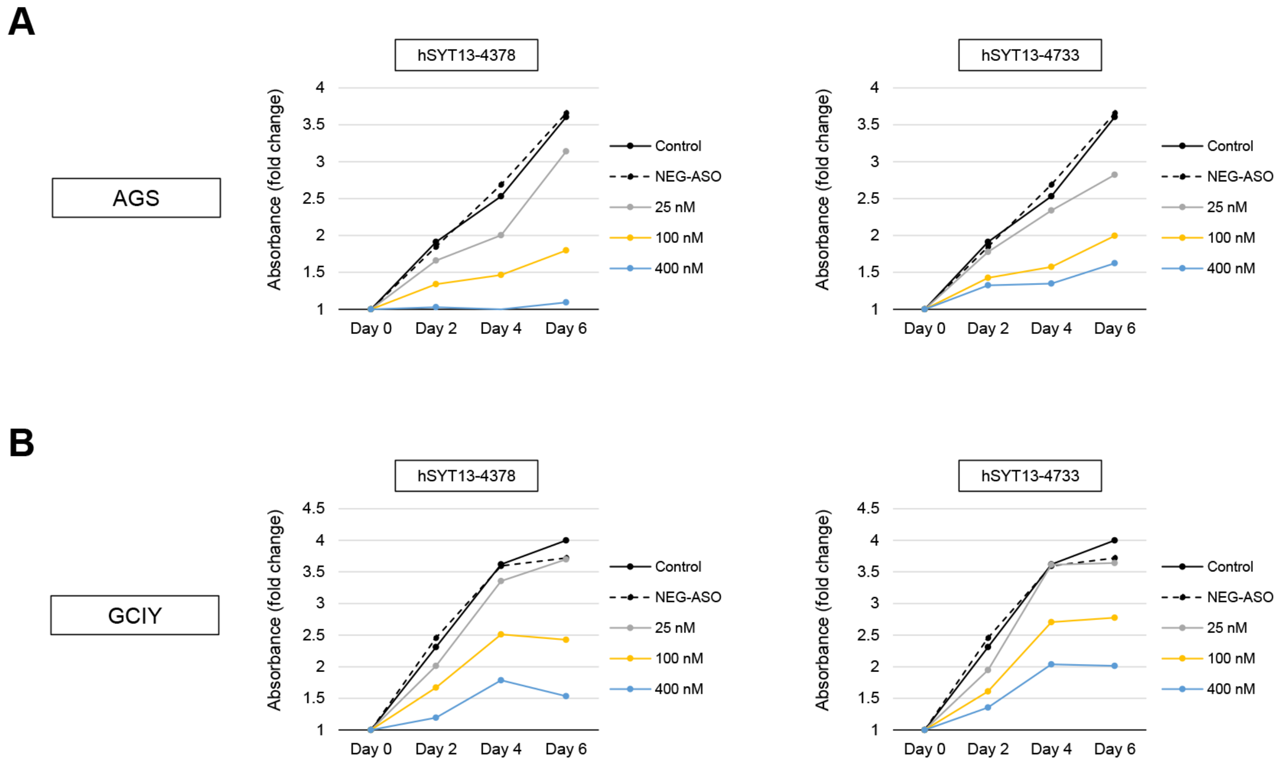

Figure S3. Concentration-dependent in vitro inhibitory effects of AmNA-modified anti-SYT13 ASOs on proliferation of AGS (A) and GCIY (B) cells.

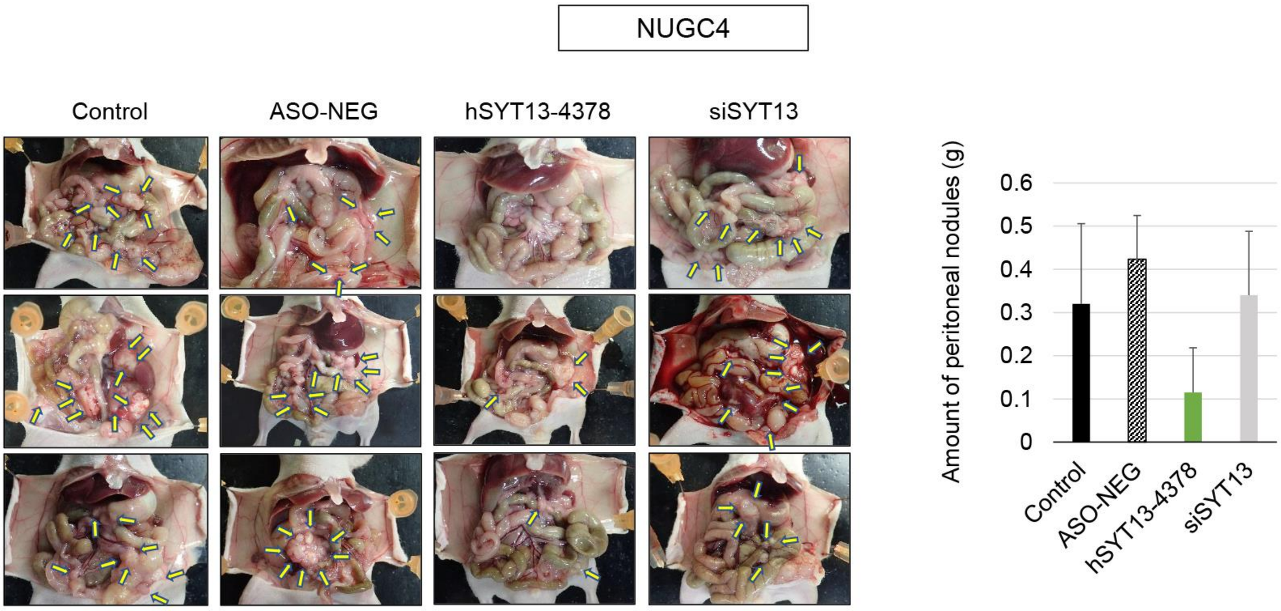

Figure S4. Therapeutic effects of intraperitoneal administration of hSYT13-4378. Macroscopic appearance of peritoneal nodules 6 weeks after implantation of NUGC4 cells and comparison of the total volume of peritoneal nodules of each treatment group.

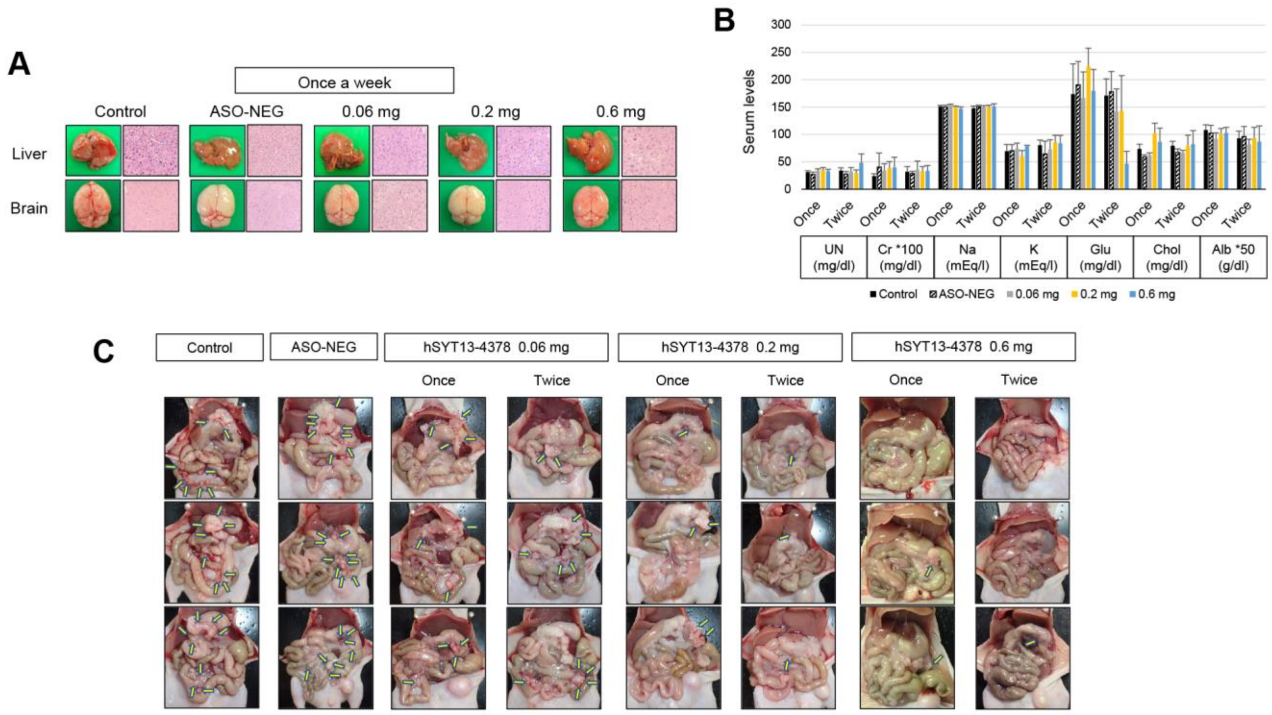

Figure S5. Toxicity to mice of intraperitoneally administered AmNA-modified anti-SYT13 ASOs. (*A*) Renal functions and metabolic parameters. (*B*) Macroscopic and microscopic findings of the brain and liver the once weekly-treated groups. (*C*) Macroscopic appearance of peritoneal nodules of each treatment group.

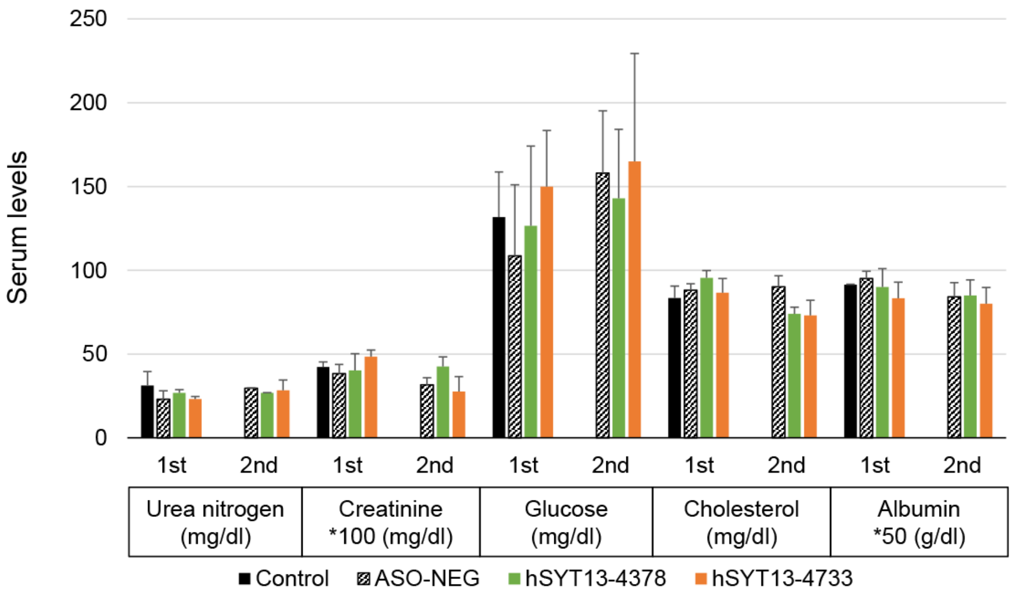

Figure S6. Blood tests after administration of 0.2 mg hSYT13-4378 or hSYT13-4733 twice weekly for 2 weeks and 2 weeks after treatment ceased.

Table S1. Sequences of 71 candidate amido-bridged nucleic acid-modified antisense oligonucleotides targeting human *SYT13* and a negative-control antisense oligonucleotide

Name	Length	Sequence
hSYT13-350-AmNA	15-mer	G(Y)^G(Y)^A(Y)^a^c^t^t^g^a^g^g^a^G(Y)^G(Y)^g
hSYT13-601-AmNA	17-mer	T(Y)^A(Y)^G(Y)^t^g^g^a^g^t^t^t^g^g^g^G(Y)^G(Y)^c
hSYT13-603-AmNA	15-mer	T(Y)^A(Y)^G(Y)^t^g^g^a^g^t^t^t^g^G(Y)^G(Y)^g
hSYT13-603-AmNA	17-mer	A(Y)^G(Y)^T(Y)^a^g^t^g^g^a^g^t^t^t^g^G(Y)^G(Y)^g
hSYT13-605-AmNA	15-mer	A(Y)^G(Y)^T(Y)^a^g^t^g^g^a^g^t^t^T(Y)^G(Y)^g
hSYT13-605-AmNA	17-mer	G(Y)^5(Y)^A(Y)^g^t^a^g^t^g^g^a^g^t^t^T(Y)^G(Y)^g
hSYT13-607-AmNA	13-mer	A(Y)^G(Y)^T(Y)^a^g^t^g^g^a^g^T(Y)^T(Y)^t
hSYT13-607-AmNA	15-mer	G(Y)^5(Y)^A(Y)^g^t^a^g^t^g^g^a^g^T(Y)^T(Y)^t
hSYT13-607-AmNA	17-mer	A(Y)^G(Y)^G(Y)^c^a^g^t^a^g^t^g^g^a^g^T(Y)^T(Y)^t
hSYT13-609-AmNA	13-mer	G(Y)^5(Y)^A(Y)^g^t^a^g^t^g^g^A(Y)^G(Y)^t
hSYT13-609-AmNA	15-mer	A(Y)^G(Y)^G(Y)^c^a^g^t^a^g^t^g^g^A(Y)^G(Y)^t
hSYT13-609-AmNA	17-mer	5(Y)^5(Y)^A(Y)^g^g^c^a^g^t^a^g^t^g^g^A(Y)^G(Y)^t
hSYT13-999-AmNA	15-mer	5(Y)^T(Y)^G(Y)^a^t^g^g^a^t^a^g^t^A(Y)^G(Y)^g
hSYT13-1000-AmNA	15-mer	G(Y)^5(Y)^T(Y)^g^a^t^g^g^a^t^a^g^T(Y)^A(Y)^g
hSYT13-1071-AmNA	15-mer	T(Y)^T(Y)^G(Y)^g^a^c^t^g^g^t^t^a^G(Y)^A(Y)^g
hSYT13-1072-AmNA	15-mer	5(Y)^T(Y)^T(Y)^g^g^a^c^t^g^g^t^t^A(Y)^G(Y)^a
hSYT13-1421-AmNA	15-mer	5(Y)^5(Y)^A(Y)^a^g^a^a^g^g^g^a^g^G(Y)^5(Y)^a
hSYT13-1614-AmNA	15-mer	A(Y)^5(Y)^A(Y)^g^a^t^g^a^g^c^a^a^A(Y)^A(Y)^t
hSYT13-1617-AmNA	15-mer	A(Y)^A(Y)^5(Y)^a^c^a^g^a^t^g^a^g^5(Y)^A(Y)^a
hSYT13-1618-AmNA	15-mer	T(Y)^A(Y)^A(Y)^c^a^c^a^g^a^t^g^a^G(Y)^5(Y)^a
hSYT13-1619-AmNA	15-mer	A(Y)^T(Y)^A(Y)^a^c^a^c^a^g^a^t^g^A(Y)^G(Y)^c
hSYT13-1622-AmNA	15-mer	T(Y)^5(Y)^A(Y)^a^t^a^a^c^a^c^a^g^A(Y)^T(Y)^g
hSYT13-1623-AmNA	15-mer	T(Y)^T(Y)^5(Y)^a^a^t^a^a^c^a^c^a^G(Y)^A(Y)^t
hSYT13-1625-AmNA	15-mer	5(Y)^5(Y)^T(Y)^t^c^a^a^t^a^a^c^a^5(Y)^A(Y)^g
hSYT13-1777-AmNA	15-mer	G(Y)^A(Y)^T(Y)^a^c^t^t^t^c^a^c^t^T(Y)^5(Y)^c
hSYT13-2631-AmNA	15-mer	A(Y)^A(Y)^G(Y)^g^c^t^c^a^t^a^a^t^T(Y)^T(Y)^a
hSYT13-2812-AmNA	15-mer	T(Y)^T(Y)^T(Y)^t^t^a^g^c^c^a^g^a^G(Y)^A(Y)^g
hSYT13-2813-AmNA	15-mer	G(Y)^T(Y)^T(Y)^t^t^t^a^g^c^c^a^g^A(Y)^G(Y)^a
hSYT13-2814-AmNA	15-mer	T(Y)^G(Y)^T(Y)^t^t^t^t^a^g^c^c^a^G(Y)^A(Y)^g
hSYT13-2815-AmNA	15-mer	T(Y)^T(Y)^G(Y)^t^t^t^t^t^a^g^c^c^A(Y)^G(Y)^a
hSYT13-3246-AmNA	15-mer	5(Y)^5(Y)^A(Y)^a^a^g^g^c^a^g^a^a^T(Y)^5(Y)^c
hSYT13-3317-AmNA	15-mer	A(Y)^A(Y)^T(Y)^t^c^t^g^t^c^t^t^a^G(Y)^G(Y)^g
hSYT13-3425-AmNA	15-mer	G(Y)^A(Y)^A(Y)^a^a^a^a^t^a^a^t^g^A(Y)^5(Y)^c
hSYT13-3426-AmNA	15-mer	A(Y)^G(Y)^A(Y)^a^a^a^a^a^t^a^a^t^G(Y)^A(Y)^c
hSYT13-4268-AmNA	15-mer	T(Y)^T(Y)^T(Y)^a^c^c^a^t^t^g^a^g^A(Y)^A(Y)^c

hSYT13-4330-AmNA	15-mer	T(Y)^A(Y)^G(Y)^g^c^a^a^a^t^a^g^t^A(Y)^A(Y)^t
hSYT13-4367-AmNA	15-mer	G(Y)^T(Y)^T(Y)^g^a^t^t^a^c^a^t^t^T(Y)^A(Y)^c
hSYT13-4368-AmNA	15-mer	T(Y)^G(Y)^T(Y)^t^g^a^t^t^a^c^a^t^T(Y)^T(Y)^a
hSYT13-4371-AmNA	15-mer	A(Y)^T(Y)^5(Y)^t^g^t^t^g^a^t^t^a^5(Y)^A(Y)^t
hSYT13-4373-AmNA	15-mer	T(Y)^5(Y)^A(Y)^t^c^t^g^t^t^g^a^t^T(Y)^A(Y)^c
hSYT13-4374-AmNA	13-mer	5(Y)^A(Y)^T(Y)^c^t^g^t^t^g^a^T(Y)^T(Y)^a
hSYT13-4374-AmNA	15-mer	T(Y)^T(Y)^5(Y)^a^t^c^t^g^t^t^g^a^T(Y)^T(Y)^a
hSYT13-4374-AmNA	17-mer	T(Y)^5(Y)^T(Y)^t^c^a^t^c^t^g^t^t^g^a^T(Y)^T(Y)^a
hSYT13-4374-AmNA	19-mer	T(Y)^5(Y)^T(Y)^c^t^t^c^a^t^c^t^g^t^t^g^a^T(Y)^T(Y)^a
hSYT13-4376-AmNA	15-mer	T(Y)^5(Y)^T(Y)^t^c^a^t^c^t^g^t^t^G(Y)^A(Y)^t
hSYT13-4376-AmNA	17-mer	T(Y)^5(Y)^T(Y)^c^t^t^c^a^t^c^t^g^t^t^G(Y)^A(Y)^t
hSYT13-4377-AmNA	15-mer	5(Y)^T(Y)^5(Y)^t^t^c^a^t^c^t^g^t^T(Y)^G(Y)^a
hSYT13-4378-AmNA	15-mer	T(Y)^5(Y)^T(Y)^c^t^t^c^a^t^c^t^g^T(Y)^T(Y)^g
hSYT13-4378-AmNA	17-mer	A(Y)^T(Y)^T(Y)^c^t^c^t^t^c^a^t^c^t^g^T(Y)^T(Y)^g
hSYT13-4380-AmNA	15-mer	A(Y)^T(Y)^T(Y)^c^t^c^t^t^c^a^t^c^T(Y)^G(Y)^t
hSYT13-4380-AmNA	17-mer	A(Y)^T(Y)^A(Y)^t^t^c^t^c^t^t^c^a^t^c^T(Y)^G(Y)^t
hSYT13-4381-AmNA	15-mer	T(Y)^A(Y)^T(Y)^t^c^t^c^t^t^c^a^t^5(Y)^T(Y)^g
hSYT13-4382-AmNA	15-mer	A(Y)^T(Y)^A(Y)^t^t^c^t^c^t^t^c^a^T(Y)^5(Y)^t
hSYT13-4382-AmN	17-mer	T(Y)^T(Y)^A(Y)^t^a^t^t^c^t^c^t^t^c^a^T(Y)^5(Y)^t
hSYT13-4716-AmNA	15-mer	5(Y)^5(Y)^5(Y)^g^a^t^t^t^t^c^t^a^T(Y)^5(Y)^c
hSYT13-4717-AmNA	15-mer	T(Y)^5(Y)^5(Y)^c^g^a^t^t^t^t^c^t^A(Y)^T(Y)^c
hSYT13-4725-AmNA(	13-mer	G(Y)^5(Y)^A(Y)^g^a^c^t^c^c^c^G(Y)^A(Y)^t
hSYT13-4725-AmN	15-mer	G(Y)^G(Y)^G(Y)^c^a^g^a^c^t^c^c^c^G(Y)^A(Y)^t
hSYT13-4725-AmN	17-mer	G(Y)^A(Y)^G(Y)^g^g^c^a^g^a^c^t^c^c^c^G(Y)^A(Y)^t
hSYT13-4727-AmNA	13-mer	G(Y)^G(Y)^G(Y)^c^a^g^a^c^t^c^5(Y)^5(Y)^g
hSYT13-4727-AmNA	15-mer	G(Y)^A(Y)^G(Y)^g^g^c^a^g^a^c^t^c^5(Y)^5(Y)^g
hSYT13-4727-AmNA	17-mer	A(Y)^T(Y)^G(Y)^a^g^g^g^c^a^g^a^c^t^c^5(Y)^5(Y)^g
hSYT13-4729-AmNA	15-mer	A(Y)^T(Y)^G(Y)^a^g^g^g^c^a^g^a^c^T(Y)^5(Y)^c
hSYT13-4778-AmNA	15-mer	G(Y)^5(Y)^T(Y)^c^a^a^c^a^a^a^t^a^G(Y)^A(Y)^t
hSYT13-4779-AmNA	15-mer	T(Y)^G(Y)^5(Y)^t^c^a^a^c^a^a^a^t^A(Y)^G(Y)^a
hSYT13-4729-AmNA	17-mer	T(Y)^A(Y)^A(Y)^t^g^a^g^g^g^c^a^g^a^c^T(Y)^5(Y)^c
hSYT13-4731-AmNA	15-mer	T(Y)^A(Y)^A(Y)^t^g^a^g^g^g^c^a^g^A(Y)^5(Y)^t
hSYT13-4731-AmNA	17-mer	A(Y)^T(Y)^T(Y)^a^a^t^g^a^g^g^g^c^a^g^A(Y)^5(Y)^t
hSYT13-4733-AmNA	17-mer	A(Y)^G(Y)^A(Y)^t^t^a^a^t^g^a^g^g^g^c^A(Y)^G(Y)^a
hSYT13-4951-AmNA	15-mer	5(Y)^A(Y)^5(Y)^a^t^t^t^t^a^c^c^c^A(Y)^G(Y)^g
hSYT13-4952-AmNA	15-mer	5(Y)^5(Y)^A(Y)^c^a^t^t^t^t^a^c^c^5(Y)^A(Y)^g
NEG-ASO	15-mer	5(Y)^A(Y)^5(Y)^a^g^t^a^t^c^t^a^t^G(Y)^T(Y)^a

Table S2. Genes at potential risk of off-target effects of amido-bridged nucleic acid-modified antisense oligonucleotides targeting human *SYT13*

**Antisense oligonucleotides**	**Off-target result**	**mRNA accession number**	**Transcription product**
hSYT13-4378	Full match	Not identified	
	1 mismatch	NM_003772.3	JRK like, transcript variant 1
		NR_104262.1	cell division cycle and apoptosis regulator 1, transcript variant 4
	2 mismatches	NM_000189.4	hexokinase 2
		NM_001009612.2	chromosome 20 open reading frame 202
		NM_001035.2	ryanodine receptor 2
		NM_001145365.1	zinc finger protein 652, transcript variant 1
		NM_001247997.1	CAP-Gly domain containing linker protein 1, transcript variant 3
		NM_001252613.1	zinc finger protein 638, transcript variant 4
		NM_001286206.1	centrosomal protein 162, transcript variant 2
		NM_001999.3	fibrillin 2
		NM_005546.3	IL2 inducible T cell kinase
		NM_006726.4	LPS responsive beige-like anchor protein, transcript variant 2
		NM_018313.4	polybromo 1, transcript variant 2
		NM_024636.3	STEAP4 metalloreductase, transcript variant 1
		NM_130759.3	GTPase, IMAP family member 1
		NM_173493.2	PAS domain containing repressor 1
		NR_027902.1	long intergenic non-protein coding RNA 303
		NR_033857.1	BMS1 pseudogene 21
		NR_037804.1	NPHP3-ACAD11 readthrough
		XM_011534442.1	CREB3 regulatory factor, transcript variant X3
		XR_245956.1	nuclear receptor subfamily 2 group C member 1, transcript variant X10
hSYT13-4733	Full match	Not identified	
	1 mismatch	Not identified	
	2 mismatches	XR_429721.2	pyruvate dehydrogenase phosphatase regulatory subunit, transcript variant X10

Table S3. Antibodies used for digital western blot imaging analyses

**Target**	**Predicted size (kDa)**	**Antibody type**	**Company**	**Catalog number**	**Dilution**
SYT13	47	Rabbit polyclonal	Aviva Systems Biology Corporation	OAAB02896	1:50
FAK	125	Rabbit monoclonal	Cell Signaling Technology	#71433	1:50
p-FAK	125	Rabbit monoclonal	Cell Signaling Technology	#8556	1:50
AKT	60	Rabbit monoclonal	Cell Signaling Technology	#4691	1:50
p-AKT	60	Rabbit monoclonal	Cell Signaling Technology	#4060	1:50
GSK3B	46	Rabbit monoclonal	Cell Signaling Technology	#12456	1:50
p-GSK3B	46	Rabbit monoclonal	Cell Signaling Technology	#5558	1:50
JAK2	125	Rabbit monoclonal	Cell Signaling Technology	#3230	1:50
p-JAK2	125	Rabbit monoclonal	Cell Signaling Technology	#3776	1:50
YAP	65-75	Rabbit monoclonal	Cell Signaling Technology	#14074	1:50
p-YAP(ser109)	78	Rabbit monoclonal	Cell Signaling Technology	#46931	1:50
Bad	23	Rabbit monoclonal	Cell Signaling Technology	#9239	1:50
p-Bad	23	Rabbit monoclonal	Cell Signaling Technology	#5284	1:50
BIRC5	16	Rabbit monoclonal	Cell Signaling Technology	#2808	1:50
CDK2	33	Rabbit monoclonal	Cell Signaling Technology	#2546	1:50
p-CDK2	33	Rabbit monoclonal	Cell Signaling Technology	#2561	1:50
b-actin	42	Mouse monoclonal	Abcam	ab8224	1:50

Supplemental Materials and Methods

Clinical Significance of *SYT13* Expression

Immunohistochemical (IHC) analysis of the in situ location and expression patterns of SYT13 was performed using a rabbit polyclonal antibody raised against SYT13 (OAAB02896; Aviva Systems Biology Corporation, San Diego, CA, USA) diluted 1: 100 in antibody diluent (Dako, Glostrup, Denmark) to probe 40 formalin-fixed, paraffin-embedded sections of well-preserved tissues from patients with pT4a gastric cancer who underwent curative gastrectomy, as previously described.[1] The staining intensity of SYT13 was categorized as negative or positive according to the percentage of stained cells in a gastric cancer component (negative, 0%–20%; positive, >20%).[2, 3] To avoid subjectivity, specimens were randomized and coded by two independent observers masked to the status of the samples.

External-validation cohorts were generated using global cohort data obtained from The Cancer Genome Atlas (TCGA) Research Network via the open source c-BioPortal (https://www.cbioportal.org/)[4] and the Kaplan–Meier Plotter (http://kmplot.com/analysis/).[4]

Cell Lines

The gastric cancer cell lines MKN1 (JCRB1433), MKN74 (JCRB0255), NUGC4 (JCRB0834), and GCIY (JRCB0555) were obtained from the Japanese Collection of Research Bioresources Cell Bank

(JCRB, Osaka, Japan). The AGS (CRL1739), KATO-III (HTB103) and N87 (CRL5822) cell lines were acquired from the American Type Culture Collection (ATCC, Manassas, VA, USA). The cell lines were tested using the short tandem-repeat PCR method and authenticated by the JCRB before the study commenced (June 2015). Cell lines were cultured at 37 °C in RPMI 1640 medium (Sigma–Aldrich) supplemented with 10% fetal bovine serum (FBS) in an atmosphere containing 5% CO_2.

Design and Synthesis of AmNA-modified Anti-SYT13 ASOs

The loop structure of *SYT13* mRNA, to which ASOs binds with high affinity, was predicted using RNAfold (http://rna.tbi.univie.ac.at//cgi-bin/RNAWebSuite/RNAfold.cgi) and mfold (http://unafold.rna.albany.edu/?q=mfold).[5] Effective sequences specifically targeting human *SYT13* mRNA were selected in consideration of homology with mus musculus for the analysis of in vivo toxicity. We designed AmNA-modified ASOs to exclude the sequences TCC and TGC, which may bind hepatocellular proteins to cause hepatotoxicity.[6, 7] CpG, which strongly induces strong inflammatory responses, was avoided as well.[8] AmNA-modified ASOs were synthesized and purified by Gene Design, Inc. using an automated DNA synthesizer (Osaka, Japan).

In Vitro Transfection of ASOs and siRNA

Transfection of ASOs or siRNA into gastric cancer cell lines was performed using the CEM method. Cells were cultured in a 24-well plate (5000 cells per well) and transiently transfected the next day

with AmNA-modified ASOs (6.25–400 nmol/L) or siRNAs specific for *SYT13* (100 nmol/L (A-014082-13 and A-014082-14) (Accell siRNA; GE Healthcare Dharmacon, Lafayette, CO, USA) in the presence of 9 mM $CaCl_2$. After transfection, cells were cultured in medium with 10% FBS for 24 h. The efficacy of inhibition of *SYT13* mRNA expression was evaluated using a quantitative real-time reverse-transcription PCR (qRT-PCR) assay with a StepOnePlus Real-Time PCR System (Applied Biosystems, Foster City, CA, USA). The *SYT13* primers were as follows: sense 5′-TGGTGGTGCTGATTAAAGCC -3′ and antisense 5′- TGCTTCTTCTTCAGCTTCCG -3′. Glyceraldehyde-3-phosphate dehydrogenase (*GAPDH*) mRNA served as an endogenous control.

Assays of Cell Function

A Cell Counting Kit-8 (Dojindo Molecular Technologies, Inc., Kumamoto, Japan) was used to analyze cell proliferation. Wound-healing assays were performed to evaluate cell migration, and cell invasiveness was assessed using BioCoat Matrigel invasion chambers (BD Biosciences, Bedford, MA, USA). We used the CytoSelect 48-Well Cell Adhesion Assay (Cell Biolabs, Inc., San Diego, CA, USA) to measure the adherence of cells to extracellular matrix components. These assays were performed as previously described.[9, 10] To evaluate total caspase activity, a Muse MultiCaspase Kit (Merck Millipore, Billerica, MA, USA) was used, and data analysis was performed using a Muse Cell Analyzer (Merck Millipore). The activities of caspase-3, 8, 9, and 12 were measured using the Caspase Colorimetric Assay Kit (BioVision, Milpitas, CA, USA). Mitochondrial membrane potential

and cell-cycle distribution were assessed using a Muse MitoPotential Kit (Merck Millipore) and a Muse Cell Cycle Kit (Merck Millipore), respectively.

Assays of Cancer Cell Stemness

Aldehyde dehydrogenase (ALDH), a surrogate marker of stem/progenitor cells, was estimated using the ALDEFLOUR fluorescent reagent system (Stem Cell Technologies, Vancouver, BC, Canada). Briefly, gastric cancer cells (10^5/ml) were suspended in ALDEFLOUR buffer. The ALDH inhibitor N,N-diethylaminobenzaldehyde (25 μM) was added to cells that were then incubated with the activated ALDEFLOUR substrate boron-dipyrromethene aminoacetaldehyde (1.5 μM) for 45 min at 37 °C. The proportion of ALDH-positive cells was determined using a FACS Calibur system (BD Biosciences, Franklin Lakes, NJ, USA), and data were analyzed using CellQuestPro software (BD Biosciences). The three-dimensional-spheroid cultures were analyzed using PrimeSurface96U multiwell plates (Sumitomo Bakelite, Tokyo, Japan). MKN1 cells were mock-transfected or transfected with a nontargeting control ASO (ASO-NEG) or AmNA-modified anti-SYT13 ASOs (hSYT13-4378 and hSYT13-4733) and incubated for 48 h. Each well contained 5000 cells. After incubation for 72 h, images of spheroids were acquired using an FSX100 (Olympus, Tokyo, Japan) fluorescence microscope.

***SYT13* Expression Vector**

SYT13 cDNAs were ligated to the pFN21A HaloTag CMV Flexi Vector (Promega, Madison, WI, USA). The *SYT13* vector (0.2 mg) was used to transfect MKN74 cells (1×10^5) using the NEON Transfection System (Thermo Fisher Scientific, Waltham, MA, USA).

Microarray and In Silico Analysis of Candidate Genes for Potential Off-target Effects of AmNA-modified Anti-SYT13 ASOs

Total RNAs extracted from NUGC4 cells transfected with mock, ASO-NEG, or AmNA-modified anti-SYT13 ASOs (hSYT13-4378 and hSYT13-4733) were subjected to microarray analysis using a 3D-Gene microarray (Toray, Tokyo, Japan). Global gene expression data were selected to evaluate potential off-target effects of AmNA-modified Anti-SYT13 ASOs as follows: 1) ≥2-fold increase or ≤0.5-fold decrease compared with mock-transfected cells and 2) no significant changes (>0.5-fold and <2-fold, respectively) in the ASO-NEG group compared with the mock group. Sequence searches allowing for mismatches, insertions, or deletions were performed using GGGenome (https ://GGGenome.dbcls.jp/), because BLAST software may overlook potential complementary regions.[11, 12] Finally, the results of microarray and in silico analyses were combined to identify candidate genes subject to potential off-target effects of AmNA-modified Anti-*SYT13* ASOs.

Intracellular Signaling Mediated by *SYT13*

SYT13 is a single-pass membrane protein. We therefore investigated the effects on *SYT13* expression

of five candidate ligands that promote peritoneal metastasis of gastric cancer. Recombinant human amphiregulin (262-AR-100), C-X-C motif chemokine ligand 12 (CXCL12, 350-NS-010), delta like canonical notch ligand 1 (1818-DL-050), heparin binding EGF like growth factor (HB-EGF, 259-HE-050), and jagged canonical notch ligand 1 (1277-JG-050) were obtained from R&D Systems (Minneapolis, MN, USA).[13-16] Each protein (0, 1, 10, 100, or 1000 ng/ml) was added to MKN1 cells (5000 cells per well), and the levels of *SYT13* mRNA were determined after incubation for 72 h.

We employed two array systems to investigate intracellular signaling pathways. The Human XL Oncology Array Kit (R&D Systems, Minneapolis, MN, USA) was used to determine the relative levels of 84 human cancer-related proteins expressed by ASO-transfected NUGC4 cells in the presence and absence of *SYT13* expression. Phosphorylation of 1006 unique sites among 409 proteins in these cells, representing the AKT, MAPK, NF-κB, and JAK/STAT signaling pathways, were quantified using the PTMScan Direct Multi-Pathway Enrichment Kit (Cell Signaling Technology, Danvers, MA, USA).[17] The associations of the PI3K signal transduction pathway, represented by activation of AKT, were analyzed using a Muse PI3K Activation Dual Detection Kit (Merck Millipore). Protein expression and phosphorylation were assessed using a capillary electrophoresis method a Wes automated system (ProteinSimple, San Jose, CA, USA) according to the manufacturer’s instructions. Digital imaging of western blot data was performed using Compass for SW version 4.0.0 analysis software (ProteinSimple). Antibodies used for this purpose are listed in Table S3.

Mouse Model of Peritoneal Metastasis

Experiments using animals were performed according to the ARRIVE guidelines and were approved by the Animal Research Committee of Nagoya University (approval number 31370).[2] First, we evaluated the effects of intraperitoneal administration of AmNA-modified anti-*SYT13* ASOs compared with those of siRNAs. MKN1 or NUGC4 cells (1×10^6 each) stably expressing luciferase were implanted into the abdominal cavities of BALBc$^{nu/nu}$ mice (males, 8 weeks old). Mice (n = 4, each condition) were intraperitoneally injected twice after implantation each week for 6 weeks with 500 µL of glucose, 0.2 mg (approximately 10 mg/kg) of ASO-NEG, and 0.2 mg of hSYT13-4378 or 0.2 mg of siRNA, in the presence of 15 mM $CaCl_2$. The mice were killed 6 weeks after implantation, and the total volume of peritoneal nodules was measured at laparotomy. We used an In Vivo Imaging System (IVIS) Lumina (Xenogen, Alameda, CA, USA) to noninvasively monitor the burden of peritoneal metastasis, as previously described.[10]

Next, the effects of AmNA-modified anti-*SYT13* ASOs on survival was evaluated after implantation of 2×10^6 NUGC4 cells into the abdominal cavities of BALBc$^{nu/nu}$ mice (males, 8 weeks old). Mice (n = 8, each condition) were intraperitoneally injected twice each week for 12 weeks postimplantation with 500 µL of glucose, 0.2 mg (approximately 10 mg/kg) of ASO-NEG, 0.2 mg of hSYT13-4378, or 0.2 mg of hSYT13-4733, in the presence of 15 mM $CaCl_2$. One satellite mouse was allocated from each treatment group to observe macroscopic findings 8 weeks after

implantation.

Toxicity of Intraperitoneal Administration of AmNA-modified Anti-SYT13 ASOs

To assess the effects of ASOs on a mouse model of intraperitoneal metastasis, 1.5×10^6 NUGC4 cells were implanted into the abdominal cavities of BALBc$^{nu/nu}$ mice (males, 8 weeks old). Mice were allocated into the two groups, which were injected once or twice each week, respectively. Mice (n = 8, each condition) were intraperitoneally injected with 500 μL of glucose, 0.2 mg (approximately 10 mg/kg) of ASO-NEG, 0.06 mg (approximately 3 mg/kg) of hSYT13-4378, 0.2 mg (approximately 10 mg/kg) of hSYT13-4378, or 0.6 mg (approximately 30 mg/kg) of hSYT13-4378, in the presence of 15 mM $CaCl_2$ for 4 weeks after implantation.

To assess the safety of intraperitoneal administration of ASOs, the appearance of the skin, food consumption, and the body weights of each group were monitored. The mice were killed 4 weeks after implantation, and data were acquired as follows: total volume of peritoneal nodules, *SYT13* mRNA levels in peritoneal nodules, blood tests, and macroscopic and pathological characteristics of the liver, kidney and brain.

We next assessed whether the liver damage caused by intraperitoneal administration of ASOs was reversible. BALBc$^{nu/nu}$ mice (males, 8 weeks old, n = 4, each condition) were intraperitoneally injected twice each week for 2 weeks after administration of 500 μL of glucose, 0.2 mg (approximately 10 mg/kg) of AmNA-modified Anti-*SYT13* ASOs (hSYT13-4378 and hSYT13-

4733), in the presence of 15 mM $CaCl_2$. Blood tests were performed 2 weeks after initiation of treatment and 2 weeks after cessation of treatment.

Statistical Analysis

The Mann–Whitney test was used to compare the differences between two groups. Kaplan–Meier curves and the log-rank test were used to analyze survival. JMP 14 software (SAS Institute Inc., Cary, NC, USA) was used for statistical analyses, and $P < 0.05$ indicates a statistically significant difference.

References

1 Kanda M, Tanaka H, Shimizu D, Miwa T, Umeda S, Tanaka C, Kobayashi D, Hattori N, Suenaga M, Hayashi M, et al. (2018). SYT7 acts as a driver of hepatic metastasis formation of gastric cancer cells. Oncogene *37*, 5355-5366.

2 Kanda M, Shimizu D, Tanaka H, Tanaka C, Kobayashi D, Hayashi M, Takami H, Niwa Y, Yamada S, Fujii T, et al. (2018). Synaptotagmin XIII expression and peritoneal metastasis in gastric cancer. Br J Surg *105*, 1349-1358.

3 Pirog EC. (2015). Immunohistochemistry and in situ hybridization for the diagnosis and classification of squamous lesions of the anogenital region. Semin Diagn Pathol *32*, 409-418.

4 Szasz AM, Lanczky A, Nagy A, Forster S, Hark K, Green JE, Boussioutas A, Busuttil R, Szabo A, Gyorffy B. (2016). Cross-validation of survival associated biomarkers in gastric cancer using transcriptomic data of 1,065 patients. Oncotarget *7*, 49322-49333.

5 Migawa MT, Shen W, Wan WB, Vasquez G, Oestergaard ME, Low A, De Hoyos CL, Gupta R, Murray S, Tanowitz M, et al. (2019). Site-specific replacement of phosphorothioate with alkyl phosphonate linkages enhances the therapeutic profile of gapmer ASOs by modulating interactions with cellular proteins. Nucleic Acids Res *47*, 5465-5479.

6 Burdick AD, Sciabola S, Mantena SR, Hollingshead BD, Stanton R, Warneke JA, Zeng M, Martsen E, Medvedev A, Makarov SS, et al. (2014). Sequence motifs associated with hepatotoxicity of locked nucleic acid--modified antisense oligonucleotides. Nucleic Acids Res *42*, 4882-4891.

7 Harada T, Matsumoto S, Hirota S, Kimura H, Fujii S, Kasahara Y, Gon H, Yoshida T, Itoh T, Haraguchi N, et al. (2019). Chemically Modified Antisense Oligonucleotide Against ARL4C Inhibits Primary and Metastatic Liver Tumor Growth. Mol Cancer Ther *18*, 602-612.

8 Ohto U, Shibata T, Tanji H, Ishida H, Krayukhina E, Uchiyama S, Miyake K, Shimizu T. (2015). Structural basis of CpG and inhibitory DNA recognition by Toll-like receptor 9. Nature *520*, 702-705.

9 Kanda M, Shimizu D, Fujii T, Sueoka S, Tanaka Y, Ezaka K, Takami H, Tanaka H, Hashimoto R, Iwata N, et al. (2016). Function and diagnostic value of Anosmin-1 in gastric cancer progression. Int J Cancer *138*, 721-730.

10 Kanda M, Shimizu D, Tanaka H, Tanaka C, Kobayashi D, Hayashi M, Iwata N, Niwa Y, Yamada S, Fujii T, et al. (2018). Significance of SYT8 For the Detection, Prediction, and Treatment of Peritoneal Metastasis From Gastric Cancer. Ann Surg *267*, 495-503.

11 Naito Y, Bono H. (2012). GGRNA: an ultrafast, transcript-oriented search engine for genes and transcripts. Nucleic Acids Res *40*, W592-596.

12 Yoshida T, Naito Y, Yasuhara H, Sasaki K, Kawaji H, Kawai J, Naito M, Okuda H, Obika S, Inoue T. (2019). Evaluation of off-target effects of gapmer antisense oligonucleotides using human cells. Genes Cells *24*, 827-835.

13 Busser B, Sancey L, Brambilla E, Coll JL, Hurbin A. (2011). The multiple roles of amphiregulin in human cancer. Biochim Biophys Acta *1816*, 119-131.

14 Huang CC, Cheng SH, Wu CH, Li WY, Wang JS, Kung ML, Chu TH, Huang ST, Feng CT, Huang SC, et al. (2019). Delta-like 1 homologue promotes tumorigenesis and epithelial-mesenchymal transition of ovarian high-grade serous carcinoma through activation of Notch signaling. Oncogene *38*, 3201-3215.

15 Izumi D, Ishimoto T, Miyake K, Sugihara H, Eto K, Sawayama H, Yasuda T, Kiyozumi Y, Kaida T, Kurashige J, et al. (2016). CXCL12/CXCR4 activation by cancer-associated fibroblasts promotes integrin beta1 clustering and invasiveness in gastric cancer. Int J Cancer *138*, 1207-1219.

16 Yasumoto K, Yamada T, Kawashima A, Wang W, Li Q, Donev IS, Tacheuchi S, Mouri H, Yamashita K, Ohtsubo K, et al. (2011). The EGFR ligands amphiregulin and heparin-binding egf-like growth factor promote peritoneal carcinomatosis in CXCR4-expressing gastric cancer. Clin Cancer Res *17*, 3619-3630.

17 Kanda M, Shimizu D, Sawaki K, Nakamura S, Umeda S, Miwa T, Tanaka H, Tanaka C, Hayashi M, Iguchi Y, et al. (2020). Therapeutic monoclonal antibody targeting of neuronal pentraxin receptor to control metastasis in gastric cancer. Mol Cancer *19*, 131.
